# Autophagy, Pyroptosis, and Ferroptosis: New Regulatory Mechanisms for Atherosclerosis

**DOI:** 10.3389/fcell.2021.809955

**Published:** 2022-01-13

**Authors:** Lin Lin, Mu-Xin Zhang, Lei Zhang, Dan Zhang, Chao Li, Yun-lun Li

**Affiliations:** ^1^ Chinese Medicine Innovation Research Institute, Shandong University of Traditional Chinese Medicine, Jinan, China; ^2^ The First Clinical Medical College, Shandong University of Traditional Chinese Medicine, Jinan, China; ^3^ College of Traditional Chinese Medicine, Shandong University of Traditional Chinese Medicine, Jinan, China; ^4^ Department of Cardiovascular, Affiliated Hospital of Shandong University of Traditional Chinese Medicine, Jinan, China

**Keywords:** atherosclerosis, cell death, autophagy, pyroptosis, ferroptosis

## Abstract

Atherosclerosis is a chronic inflammatory disorder characterized by the gradual buildup of plaques within the vessel wall of middle-sized and large arteries. The occurrence and development of atherosclerosis and the rupture of plaques are related to the injury of vascular cells, including endothelial cells, smooth muscle cells, and macrophages. Autophagy is a subcellular process that plays an important role in the degradation of proteins and damaged organelles, and the autophagy disorder of vascular cells is closely related to atherosclerosis. Pyroptosis is a proinflammatory form of regulated cell death, while ferroptosis is a form of regulated nonapoptotic cell death involving overwhelming iron-dependent lipid peroxidation. Both of them exhibit distinct features from apoptosis, necrosis, and autophagy in morphology, biochemistry, and genetics. However, a growing body of evidence suggests that pyroptosis and ferroptosis interact with autophagy and participate in the development of cancers, degenerative brain diseases and cardiovascular diseases. This review updated the current understanding of autophagy, pyroptosis, and ferroptosis, finding potential links and their effects on atherogenesis and plaque stability, thus providing ways to develop new pharmacological strategies to address atherosclerosis and stabilize vulnerable, ruptured plaques.

## 1 Introduction

Atherosclerosis, caused by the accumulation of low-density lipoprotein (LDL) in the subendothelial matrix, is a progressive disease characterized by endothelial damage, inflammatory cell infiltration, cell proliferation, and fat deposition (Lusis, 2000). Proinflammatory and anti-inflammatory mechanisms, along with inadequate cellular inflammation resolution, are the initial factors that promote and accelerate the process of atherosclerotic plaque formation (Back et al., 2019). The composition and vulnerability of a plaque play a principally decisive role in plaque stability, thrombosis, and thrombus-mediated acute coronary events. The functional status, survival and death of vascular cells, including endothelial cells (ECs), vascular smooth muscle cells (VSMCs), and macrophages, can affect the formation and stability of plaques, thus affecting the progression of atherosclerosis.

The endothelial lining of lesion-prone areas of the arterial vasculature is highly susceptible to risk factors associated with atherosclerosis. Oxidized LDL (ox-LDL) is recognized by pattern-recognition receptors in ECs and induces endothelial dysfunction by triggering a cascade of oxidative stress and inflammatory responses (Marchio et al., 2019). Moreover, hyperlipidemia stimulates EC activation before monocyte recruitment (Yin et al., 2015) and activated ECs promote the infiltration of inflammatory cells into atherosclerotic lesions by increasing the release of adhesion molecules, resulting in plaque aggregation and eventually atherosclerosis (Libby et al., 2011). The subendothelial accumulation of foam cells is a major hallmark of early atherosclerotic lesions. It has been recently found that more than 50% of foam cells in plaques are contributed by VSMCs (Allahverdian et al., 2014; Wang Y. et al., 2019), which is the major constituent of the fibrous cap (Pan and Reilly, 2019). Therefore, the death of VSMCs and VSMCs-derived foam cells decreases lesion cellularity, weakens the fibrous cap of the plaque, and increases plaque instability (Clarke et al., 2010). Advanced plaques contain many macrophages with a proinflammatory phenotype, which secrete stromal degradation enzymes, growth factors, cytokines, and intracellular lipids into the extracellular space, leading to plaque instability, plaque rupture, and thrombotic events. Moreover, in advanced atherosclerotic plaques, up to 50% of dead cells are macrophages; the mechanisms of macrophage death involve the decay of lesion cellularity and the promotion of inflammation. Macrophage death is also a significant feature of advanced plaques in atherosclerosis and serves as a catalyst for the formation of necrotic core and plaque instability (Lutgens et al., 1999; Martinet et al., 2019). Atherosclerosis is still the leading cause of death worldwide because of the acute occlusion caused by the formation of a thrombus or blood clot. Hence, the different processes and mechanisms involved in plaque stabilization need to be further explored to find new therapies for atherosclerosis.

Regulated cell death (RCD) is a highly regulated cellular response that controls cell fate in multicellular organisms after they are subjected to various cellular pressures and/or external stimuli. The most common modulated form of cell death is called apoptosis (Kerr et al., 1972); other forms of RCD have been gradually discovered, such as autophagy (Ashford and Porter, 1962), pyroptosis (Fink and Cookson, 2005), and ferroptosis (Dixon et al., 2012). Different types of RCD display distinct features, meanwhile sharing many similar characteristics with considerable overlap and crosstalk (Table 1). The interactions between these cell death programs play a role in controlling the ultimate outcome during cell death (Lockshin and Zakeri, 2004). Under normal circumstances, various forms of RCD remove damaged or infected cells from the affected tissues so that the surrounding healthy cells can better perform their proper functions. Nevertheless, the loss of control over single or mixed types of RCD can lead to human diseases, such as cancer, neurodegeneration, autoimmune diseases, infectious diseases, and cardiovascular diseases. Multiple types of RCD have been identified in the pathological process of atherosclerosis, but the intricate overlapping effects and interactions have rarely been systematically summarized. Based on this, the present review provided a current overview of the known signal cascade and interaction of the common patterns of RCD, namely autophagy, pyroptosis, and ferroptosis, and the latest understanding of their functional role and significance in atherosclerosis.

**TABLE 1:** The main features of autophagy, pyroptosis, and ferroptosis.

RCD	Autophagy	Pyroptosis	Ferroptosis
Morphological features	Formation of double-membraned autolysosomes	Cell swelling, pore formation, membrane rupture, massive leakage of cytoplasmic components	Cytoplasmic and organelles swelling, dysmorphic shrunken mitochondria with the reduced cristae and ruptured outer membrane, unchanged nucleus
Biochemical features	Increased lysosomal activity	Caspase-1/4/5/11 activation and proinflammatory cytokines release	Iron accumulation, lipid peroxidation
Immune features	Mostly anti-inflammatory	Proinflammatory	Proinflammatory
Key genes	ATG5, ATG7, ATG12, TFEB	NLPR3, caspase-1/4/5/11, IL-1β, IL-18	TFR1, GXP4, Nrf2, FSP1
Key regulatory pathways	PI3K–AKT–mTOR, MAPK–ERK1/2–mTOR pathway	NLRP3–Caspase-1–GSDMD pathway, Caspase-4/5/11–GSDMD pathway	System x_c_^−^–GSH–GPX4 pathway, Nrf2–Keap1 pathway
Released DAMP	HMGB-1	IL-1β, IL-18, ATP, HMGB-1	HMGB-1
Inducers	Rapamycin, C2-ceramide, lithium, sodium, valproate	Ivermectin, ZnO-NPs	Erastin, sulfasalazine, sorafenib, BSO, RSL3, ML162, ML210, FINO_2_, FIN56, withaferin A
Inhibitors	3-MA, LY294002, Bafilomycin A1, hydroxychloroquine, PIK-III, compound 31, Vps34-ln1	Necrosulfonami-de	Deferoxamine, dexrazoxane, ferritin, FPN1, vitamin E, ferrostatin-1, liproxstatin-1
References	Klionsky and Emr (2000), Ohsumi (2001), Thi and Reiner (2012), Xie et al. (2015)	Fink and Cookson (2006), Miao et al. (2011), Boucher et al. (2018), Hoseini et al. (2018)	Seiler et al. (2008), Dixon et al. (2015), Galluzzi et al. (2018), Bersuker et al. (2019), Bao et al. (2020), Riegman et al. (2020)

## 2 The Roles of Autophagy in Atherosclerosis

### 2.1 Overview of Autophagy

Autophagy is an evolutionarily conservative homeostatic mechanism consisting of three general subtypes: macroautophagy, microautophagy and chaperone-mediated autophagy (CMA) (Klionsky et al., 2016). Macroautophagy (hereafter referred to as autophagy) is a lysosome-dependent intracellular degradation system by which cytoplasmic organelles, macromolecules, proteins, and invading pathogens are degraded in the lysosome, with the ability to produce new building blocks and energy for cellular renovation and homeostasis (Mizushima and Komatsu, 2011; Parzych and Klionsky, 2014). Morphologically, autophagy begins in the pre-autophagosomal structure in the cytoplasm and gradually develops into a phagophore, followed by an autophagosome, a double-membraned vacuole containing damaged organelles and denatured macromolecules. Subsequently, the outer membrane of autophagosome fuses with the lysosomal membrane to form autolysosome, and the inner membrane and encapsulated substances of autophagosome enter the lysosomal cavity and are degraded by activated lysosomal hydrolases (Klionsky and Emr, 2000).

Autophagy is a continuous and dynamic process that tightly regulated by autophagy-related genes (ATG) (Kang et al., 2011; Xie et al., 2015). Mammalian target of rapamycin (mTOR) is negatively involved in autophagy regulation. When the cell in the absence of growth factors and amino acids or is stimulated by rapamycin, unc-51 like autophagy-activating kinase 1 (ULK1) is separated from mTOR and undergoes rapid dephosphorylation; activated ULK1 promotes ATG13 phosphorylation and autophagy (Jung et al., 2009; Kim et al., 2011). The class III phosphatidylinositol 3-kinase (Class III PI3K) complex, which includes phosphatidylinositol 3-kinase, catalytic subunit type 3 (PIK3C3)/VPS34, Beclin-1, and ATG14, promotes the nucleation of phagophores by generating the phosphatidylinositol-3-phosphate and recruiting other factors involved in the process of autophagosome formation (Thi and Reiner, 2012; McKnight and Zhenyu, 2013; Bernard and Klionsky, 2014). Subsequently, the phagophore expands by membrane addition though two ubiquitin-like modification processes, among which ATG12-conjugation and microtubule-associated protein 1A/1B-light chain 3 (LC3)-modification play the most crucial roles (Ohsumi, 2001; Tanida et al., 2004). ATG12 is activated by ATG7, and then combines with ATG5 and ATG16L to form a ATG12-ATG5-ATG16L complex at phagophores. In the process of LC3-modification, proLC3 is first processed into LC3-I with the help of ATG4, activated by ATG7, transported to ATG3, and then processed into LC3-II, which is the membrane-bound form localized on preautophagosomes and autophagosomes. After the extension, the outer membrane of autophagosome fuses with lysosomes to form autolysosome and degrade encapsulated substances.

Autophagy plays a dual role, either preventing or promoting cell death, depending on the environment (Fitzwalter and Thorburn, 2015). On the one hand, autophagy-related stress tolerance protects cells against various kinds of cellular death stimuli. Free amino acids and fatty acids produced by the autophagy degradation of unused protein aggregates or damaged organelles can be used for protein synthesis and energy production to adapt to environmental stress (Kroemer et al., 2010). On the other hand, excessive or uncontrolled autophagy can result in the degradation of prosurvival proteins and promote cell death, termed “autophagy-dependent cell death” (Denton and Kumar, 2019). Autophagy regulates inflammatory responses and cell death pathways, thereby influencing the pathogenesis of diseases, including atherosclerosis.

### 2.2 Effects of Autophagy on Atherosclerosis

**FIGURE 1:**
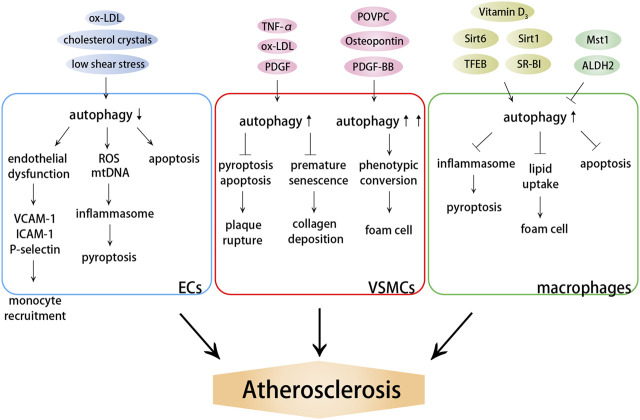
Effects of autophagy on atherosclerosis. Autophagy dysfunction induces endothelial dysfunction and inflammation, promoting monocyte recruitment and cell death. Autophagy dysfunction aggravates plaque instability and promotes the formation of foam cells by influencing VSMC death and phenotypic conversion. Autophagy dysfunction induces lipid accumulation, macrophage foam cell formation and cell death.

Autophagy is closely associated with atherosclerosis, which has been confirmed by the expression profile of ARGs (Chen Y. et al., 2021). Multiple autophagy triggers are present in atherosclerotic plaques, such as reactive oxygen species (ROS), LDL, inflammatory mediators, and tumor necrosis factor-α (TNF-α) (Shao et al., 2016; Zhu et al., 2019). Mild cellular stress, including oxidative stress, ox-LDL, and endoplasmic reticulum (ER) stress, stimulates mild adaptive autophagy of vascular cells (Menghini et al., 2014), which promotes cell survival by degrading damaged organelles and proteins, thereby protecting vascular tissues from oxidative stress or inflammation. It has been confirmed that autophagy can diminish the atherosclerotic plaque area and effectively preserve the stable plaque phenotype, including reduced lipid deposition and proinflammatory macrophages; increased anti-inflammatory macrophages, collagen content, and VSMCs; and lessened cell death within the plaques (Li et al., 2013). Nevertheless, similarly, dysfunction of autophagy has also been found to occur in the progression of atherosclerosis, regardless of the presence or absence of these autophagy stimulators in atherosclerotic plaques. The expression of autophagy markers ATG13 and LC3 in aortic intimal ECs with severe atherosclerosis was found to be significantly higher than the expression in those without atherosclerosis (Chen et al., 2017). Severe oxidative stress or inflammation stimulates excessive autophagy, leading to autophagy-dependent cell death, decreased collagen synthesis, thinning of the fibrous cap, plaque instability, lesions thrombosis, restenosis, and acute coronary events (Cai et al., 2018; Ge et al., 2021). Thus, the resistance to excessive autophagy in an early stage is critical in preventing arteriosclerosis, further leading to serious cardiovascular complications. Except excessive autophagy, the loss of autophagy is more prevalent and may be an important determinant of atherosclerosis. In addition, autophagy may also become insufficient, especially in advanced lesions with a large amount of oxidative stress. In this case, autophagy is functional, but is subsequently unable to cope with excess stress in the plaques, leading to cell apoptosis (Mollace et al., 2015). Therefore, the cytoprotective autophagic pathway may be transformed into a maladaptive pathway depending on the developmental stage of the plaques (Figure 1).

#### 2.2.1 Autophagy Dysfunction Induces Endothelial Dysfunction and Inflammation

Endothelial dysfunction, leukocyte adhesion, and foam macrophage formation are the main pathogeneses of atherosclerosis. Endothelial dysfunction, in particular, is thought to be the first step in atherosclerosis (Gimbrone and Garcia-Cardena, 2016). The known mechanisms related to atherosclerosis and endothelial dysfunction include impaired eNOS ability to produce NO, increased oxidative stress, inflammation and impaired autophagy. EC autophagy deficiency improves the levels of vascular cell adhesion molecule-1 (VCAM-1), intercellular adhesion molecule-1 (ICAM-1), von Willebrand factor, and P-selectin, thus promoting the infiltration of macrophages and foam cells, as well as increasing the risk of arterial thrombosis (Wu Q. et al., 2019; Perrotta et al., 2020). Furthermore, Vion et al. reported that specific deficiency in endothelial autophagy only stimulated the development of atherosclerotic lesions in atheroresistant areas, but not in atheroprone areas, in which the endothelial autophagic flux was already blocked (Vion et al., 2017). Thus, increasing basal autophagy and preventing the age-dependent decline in the autophagic flux are effective strategies for restoring endothelial health and inhibiting atherogenesis.

Current evidence supports the central role of EC inflammation in atherosclerosis. Oxidative stress is considered as a key signal in the progression of inflammation, and autophagy is a protective mechanism that protects plaque cells and ECs from certain stimulation on the arterial wall, especially oxidative damage (Cho and Choi, 2019). During oxidant stress, damaged mitochondrial DNA (mtDNA) that escaped autophagy resulted in a potent inflammatory response in atherosclerosis (Ding et al., 2013a). Increased autophagy significantly suppressed chronic vascular inflammation, restrained the formation of plaques, decreased plaque area, and attenuated atherogenesis (Pankratz et al., 2018; Meng Q. et al., 2021), whereas the inhibition or impairment of autophagy exacerbated the inflammation response (Guo F. X. et al., 2019). These studies implied that autophagy was a protective mechanism against endothelial inflammation and a potential target for treating atherosclerosis and related cardiovascular diseases.

Given the protective effect of autophagy on atherosclerotic resistance, the mechanisms of EC autophagy regulation in atherosclerosis have been gradually revealed. Arterial wall shear stress is considered to be one of the important factors in regulating EC autophagy. High shear stress triggers protective autophagy to limit atherosclerotic plaque formation by preventing endothelial apoptosis, senescence, and inflammation, whereas low shear stress, a potential proatherogenic factor, suppresses autophagy by activating the mTOR pathway (Vion et al., 2017; Yuan et al., 2020). The defect in endothelial autophagy may be the missing link between low shear stress and atherosclerosis. Moreover, caveolin-1 (CAV1) plays a critical role in atherogenesis by controlling NO production, vascular inflammation, and extracellular matrix remodeling (Pavlides et al., 2014; Ramirez et al., 2019). A recent study further found that CAV1 could interact with the ATG5–ATG12 complex and regulate the autophagosome formation by influencing the cellular localization of autophagosome components in lipid rafts. The increased autophagy activation and the autophagic flux of ECs caused by CAV1 deficiency mediated atheroprotection by attenuating EC activation in response to proatherogenic cytokines, inhibiting inflammation and macrophage recruitment (Zhang X. et al., 2020). Sun et al. reported that the deficiency of proprotein convertase subtilisin/kexin type 9 (PCSK9), which bound to the LDL reporter (LDLR) and thereby promoted its intracellular degradation, could affect atherogenesis by modulating the levels and properties of apolipoprotein B (ApoB)-containing lipoproteins dependent on increased autophagy signaling pathway and autophagic flux, rather on the effect of PCSK9 on cell surface LDLRs (Sun H. et al., 2018). This result revealed a critical mechanism by which PCSK9 inhibited atherosclerosis, in which autophagy was a major participant. Moreover, microRNAs (miRNAs), which have been identified as the critical regulators of gene expression in many organisms and biological processes, were studied extensively. MiR-214-3p regulates ox-LDL-initiated EC autophagy by directly targeting 3′-UTR of ATG5, which may play an appropriate role in the pathogenesis of atherosclerosis (Wang et al., 2018). Santovito et al. reported a noncanonical inhibition of caspase-3 by miR-126-5p, which was transported into the nucleus and bound to caspase-3, preventing caspase dimerization and downregulating activity to limit apoptosis. The aforementioned antiapoptotic effect conferred endothelial protection by autophagy in atherosclerosis, and the nuclear import of miR-126-5p was blocked by the ablation of ATG5 (Santovito et al., 2020), reflecting the potential effect of ECs autophagy in cell death resistance and atheroprotection.

#### 2.2.2 Autophagy Dysfunction Aggravates Plaque Instability by Influencing Vascular Smooth Muscle Cell Death and Phenotypic Conversion

Defective autophagy caused by VSMC-specific HuR knockout has recently been reported to trigger off plaque formation and plaque instability (Liu S. et al., 2021). Actually, the death of VSMCs and VSMC-derived foam cells is adverse to maintaining plaque stability, while autophagy promotes cell survival. Autophagy and mitophagy induced by oxidized lipids in the plaques protect VSMCs against apoptosis (Martinet et al., 2008; Swiader et al., 2016). In addition, several cytokines and growth factors, such as TNF-α and platelet-derived growth factor (PDGF), also promote autophagy to resist cell death (Jia et al., 2006; Salabei et al., 2013). Compared with healthy human VSMCs, VSMCs isolated from carotid plaque specimens showed increased autophagy (Yu et al., 2017). However, a fivefold decrease in LC3β expression was observed when comparing carotid plaques in symptomatic and asymptomatic patients, suggesting that decreased autophagy was associated with the clinical stage of atherosclerosis (Swaminathan et al., 2014). VSMC-specific ATG7 knockout in ApoE^−/−^ mice significantly increased the susceptibility of VSMCs to apoptotic cell death, leading to plaque growth, medial disruption and aneurysm formation, which provided the evidence that autophagy deficiency of VSMCs aggravated atherosclerosis via inducing VSMC death (Osonoi et al., 2018). ATG7 deletion also accelerated the development of premature senescence, which was characterized by increased total collagen deposition, nuclear hypertrophy, and CDKN2A-RB-mediated G1 proliferative arrest (Grootaert et al., 2015). Furthermore, VSMC autophagy in ApoE^−/−^ mice stimulated cholesterol efflux and inhibited lipid accumulation and necrotic core formation (Michiels et al., 2016), reflecting a regulatory effect of VSMC autophagy on lipid metabolism.

Phenotypic conversion promotes the migration and proliferation of VSMCs and the formation of VSMC-derived foam cells (Rong et al., 2003). Autophagy may play a critical role in the phenotypic conversion of VSMCs, as diverse stimuli promoting phenotypic conversions, such as ROS, oxidized lipids, and metabolic stress, are also associated with autophagy (Tai et al., 2016). Some autophagy-inducing stimuli, including PDGF-BB, POVPC, and osteopontin, promoted the loss of contractile phenotype and motivated VSMC proliferation and migration, whereas inhibiting autophagy could promote the maintenance of the contractile phenotype and prevent hyperproliferation (Chaulet et al., 2001; Li et al., 2012; Salabei and Hill, 2013). Furthermore, Wang et al. reported that nicotine-mediated autophagy in VSMCs triggered off phenotypic transition and accelerated atherosclerosis via the nAChRs/ROS/NF-κB signaling pathway (Wang Z. et al., 2019). Moreover, nicotine promoted the synthesis and secretion of cathepsin S that stimulated VSMC migration by activating autophagy (Ni et al., 2020). These results reflected that nicotine-modulated autophagy disorder in VSMCs induced and aggravated atherosclerosis, revealing the potential role of smoking in VSMCs and vascular diseases through the autophagic-lysosomal machinery. In brief, autophagy served as a safeguarding mechanism against VCMCs senescence and death; however, excessive activation of autophagy might aggravate atherosclerosis by triggering a synthetic phenotype of VSMCs.

#### 2.2.3 Autophagy Dysfunction Induces Lipid Accumulation and Macrophage Foam Cell Formation

In macrophages, ox-LDL and one of its primary oxysterols, 7-ketonecholesterol, can directly or indirectly stimulate autophagy through ER stress, promoting the survival of macrophages by promoting the clearance of damaged proteins and organelles. Sergin et al. first described the functional status of the autophagy-lysosome system in murine and human atherosclerotic plaques through two key autophagy markers LC3 and SQSTM1/p62 (Sergin et al., 2017). They found that early atherosclerotic lesions had high levels of LC3 co-localized with SQSTM1, indicating the recruitment of autophagy as a stress response to burgeoning plaques. However, advanced lesions were characterized by reduced LC3 and dissociation from SQSTM1, suggesting an extensive dysfunction during the autophagic process (Sergin et al., 2017).

Autophagy of macrophages plays an important role in the pathogenesis of atherosclerosis by inhibiting oxidative stress, inflammation, and the formation of foam cells. For example, macrophage-specific overexpression of TFEB, a master transcriptional regulator of autophagy-lysosome biogenesis, induces a broad atheroprotection by reversing the autophagy dysfunction, including reduction of overall plaque burden and characteristics of plaque complexity, while the effect cannot be achieved in the absence of ATG5 or SQSTM1 (Sergin et al., 2017). Moreover, TFEB also modulates the inflammatory status and enhances the antioxidative capacity by increasing the transcription of genes encoding antioxidant factors (Lu et al., 2017). Arsenic trioxide promoted autophagy and autophagosome-lysosome fusion by triggering the nuclear translocation of TFEB, thus preventing atherosclerosis (Fang et al., 2021). MiR-761 regulates autophagy via the mTOR–ULK1 pathway and subsequently represses the production of inflammatory cytokines, as well as the formation of foam cells (Wang C. et al., 2020), reflecting the critical role of macrophage autophagy in inflammatory resistance and foam cell formation. The activation of autophagy in monocytes also prevents plaque vulnerability and subsequent plaque rupture (Cheng et al., 2015).

The formation of macrophage-derived foam cells is the initial stage of atherosclerosis. Autophagy activation of macrophages caused by sirtuin 6 (Sirt6) overexpression can restrain apoptosis, reduce macrophage foam cell formation, and stabilize atherosclerosis plaques (He et al., 2017; Wang T. et al., 2020). The upregulation of Sirt1 expression or enhanced interaction between Sirt1 and FOXO1 also promotes autophagy and subsequently modulates macrophage polarization, thus attenuating foam cell formation (Luo et al., 2020; Hui et al., 2021). Vitamin D_3_ can recover ox-LDL-impaired autophagy and attenuate the lipid accumulation in macrophages, and scavenger receptor class B type I (SR-BI) can also promote autophagy by regulating *Tfeb* expression and recruiting VPS34-Beclin-1 complex, which all inhibit the conversion of macrophages into foam cells (Kumar et al., 2021; Tao et al., 2021).

The differences in the polarization of macrophages lead to differences in the number and distribution of polarized macrophages in plaques. Classically activated (M1) and alternatively activated (M2) macrophages connect to produce atherosclerotic plaques, and M2 macrophages can suppress foam cell transformation (Moore et al., 2013). Sun et al. found that rapamycin stimulated M1 macrophages and inhibited early atherosclerosis by inducing autophagy, and FTY720 could transform foam cells into M2 macrophages through the autophagy pathway, alleviating advanced atherosclerosis (Sun R. Z. et al., 2018). Therefore, autophagy played a role in alleviating atherosclerosis by selectively removing macrophages or changing the polarization state within plaques.

The accumulation of lipid-rich macrophages is considered as a marker of plaque instability, and intact autophagic machinery is essential in limiting lipid uptake by macrophages. Macrophage-specific ATG7-deficient mice exhibited increased susceptibility to atherosclerosis by increasing macrophage LDL uptake and foam cell formation. Compared with ApoE^−/−^ mice, the suppressed autophagic flux caused by mammalian Ste20-like kinase 1 (Mst1) overexpression increased plaque area, lipid core, and macrophage accumulation (Wang et al., 2016). Furthermore, acetaldehyde dehydrogenase 2 (ALDH2) has been reported to interact with adenosine monophosphate-activated protein kinase (AMPK) and then disturb lysosomal function and autophagy, thus repressing cholesterol hydrolysis in lysosomes and promoting lipid deposition and foam cell formation. LDLR can block the effect of ALDH2 through interaction with AMPK, whereas ALDH2 rs671 mutant attenuates this interaction and further promotes the release of ALDH2 to the nucleus where ALDH2 inhibits the transcription of a lysosomal proton pump protein ATP6V0E2 (Zhong et al., 2019). This result provides a possible molecular mechanism to explain why East Asians carrying the single nucleotide polymorphism ALDH2 rs671 have an increased risk of cardiovascular disease (Han et al., 2013). To sum up, the data revealed the function of macrophage autophagy in atherosclerosis and supported several practical methods of enhancing autophagy and degradative capacity of macrophages as a therapy for atherosclerotic vascular disease.

#### 2.2.4 Effect of Lipophagy and Mitophagy in Atherosclerosis

In atherosclerosis, a majority of lipids stored in the foam cells in the form of lipid droplets consist mainly of free cholesterol and cholesterol esters. Therefore, promoting lipid droplet degradation and cholesterol efflux is an effective strategy to restrain foam cell formation and atherosclerosis process. Lipophagy prevents cellular lipid accumulation by autophagic degradation of lipid droplets via lysosomal acid lipases, thus playing a protective role in atherosclerosis (Ouimet et al., 2017). Sirt6 overexpression in foam cells accelerates cholesterol efflux via the autophagic pathway, which can be largely reversed by miR-33 (He et al., 2017). Ouimet et al. found that miR-33 restrained lipid droplet catabolism and cholesterol mobilization by repressing lipophagy. Importantly, the effect of miR-33 in regulating lipophagy lies the upstream of its effect on the ATP-binding cassette transporter A1-dependent cholesterol efflux, as miR-33 inhibitors cannot enhance the cholesterol efflux in autophagy-deficient macrophages, highlighting the importance of lipophagy in cellular lipid metabolism (Ouimet et al., 2017). Moreover, with aging and lipid accumulation, lipophagy activity decreases, leading to the excessive accumulation of lipid droplets, which further impairs lipophagy activity. Thus, improving the decreased activity of lipophagy may play a critical role in the blockage of foam cells formation and atherosclerosis development.

Aging within the aorta during normolipidemia results in mitochondrial dysfunction and an increase in the IL-6 level, accompanied by elevated mitophagy. Enhanced mitophagy pharmacologically during hyperlipidemia increased aortic mitochondrial function and inhibited atherosclerosis by removing damaged or dysfunctional mitochondria, implying that novel therapies to improve vascular mitochondrial bioenergy prior to hyperlipidemia may attenuate age-related atherosclerosis (Tyrrell et al., 2020). Furthermore, mitophagy in macrophages induced by melatonin scavenged mitochondrial ROS and subsequently repressed prolonged NLRP3 inflammasome activation, thus markedly attenuating atherosclerotic plaque area and vulnerability (Ma et al., 2018). These findings confirmed that mitophagy avails to atherosclerosis resistance. However, ox-LDL-stimulated nuclear receptor subfamily 4 group A member 1 (NR4A1) overexpression caused Parkin-mediated mitophagy through the post-transcriptional modification of Ca^2+^/calmodulin-dependent protein kinase II, while excessive mitophagy significantly consumed mitochondrial mass, resulting in energy shortage and mitochondrial dysfunction and thus aggravating endothelial apoptosis and atherosclerosis (Li P. et al., 2018).

Taken together, the dysregulation of autophagy is present in atherosclerosis. Basic autophagy protects plaque cells from oxidative stress by degrading damaged intracellular substances, whereas overstimulated or deficient autophagic flux may lead to the death of vascular cells and plaque instability in a variety of ways, including promoting inflammation and lipid accumulation. Skyrocketing researches provide insight into the regulation of autophagic homeostasis as a critical way for therapeutic intervention for atherosclerosis. However, notably, in contrast to the proatherogenic role, a counter-regulatory response of autophagy disruption occurs in dendritic cells or T cells, which maintains immune homeostasis and limits atherogenesis (Amersfoort et al., 2018; Clement et al., 2019). Therefore, different types of cell-specific autophagy regulation have different effects on the development of atherosclerosis.

## 3 The Roles of Pyroptosis in Atherosclerosis

### 3.1 Overview of Pyroptosis

Pyroptosis is a new caspase-dependent proinflammatory form of regulatory cell death, characterized by cell swelling, pore formation, and membrane rupture, resulting in the massive leakage of cytoplasmic components (Hoseini et al., 2018). Different from the immunologically silent cell death presented by apoptosis, pyroptosis is a response to pathogen-associated molecular patterns (PAMPs) derived from invading pathogens or damage-associated molecular patterns (DAMPs) induced by endogenous stress that leads to RCD and inflammatory response following the release of cytokines (Tavakoli Dargani et al., 2018). Caspases are cysteine proteases that initiate or execute cellular programs causing inflammation or cell death (Schroder and Tschopp, 2010). Pro-caspase-1 is stored in secretory lysosomes, where it awaits exocrine-induced stimulation; otherwise, these molecules may undergo lysosomal degradation (Andrei et al., 1999).

Caspase-dependent pyroptosis requires the activation of the typical inflammatory response (Xue et al., 2019). As proinflammatory caspases, the catalytic activity of caspase-1/4/5/11 is strictly regulated by signal-dependent autoactivation within multiprotein complexes called inflammasomes (Martinon et al., 2002). Inflammasomes are molecular platforms activated in response to cellular infection, toxic insults impinging on the cell, or intracellular stress releasing interleukin (IL)-1 family members, triggering the maturation of proinflammatory cytokines and inducing cell death in the form of pyroptosis (Schroder and Tschopp, 2010; Del Re et al., 2019). An important mechanism of inflammation is the induction of inflammasomes, which requires two signals: the priming signal occurs in response to receptor activation, including interferon (IFN) signaling and Toll-like receptors (TLR) ligands, and induces the transcriptional upregulation of inflammasome components via nuclear factor κB (NF-κB); then, the activating signal provided by an inflammasome activator in the form of a PAMP (e.g., Gram-positive and -negative bacteria, bacterial toxins, DNA and RNA viruses, and fungi) or DAMP (e.g., ATP, uric acid crystals, silica crystals, cholesterol crystals, saturated fatty acids, extracellular histones, lysophosphatidylcholine, mitochondrial ROS and DNA, and aluminum hydroxide) induces the assembly of the inflammasome mediated by kinase NEK7, apoptosis-associated speck-like protein (ASC), and caspase-1 (Bauernfeind et al., 2009; Mathur et al., 2018; Tang et al., 2019). Typical inflammasomes, such as NLRP1b, NLRP3, NAIP-NLRC4, AIM2, and Pyrin, have been found to activate caspase-1, ultimately inducing pyroptosis (Schroder and Tschopp, 2010).

Once activated in response to many stimuli, caspase-1 cleaves the N-terminal and C-terminal domains of the executive protein of pyroptosis, named gasdermin D (GSDMD). These peptides contain the N-terminal active domain of GSDMD, which oligomerizes on the cytoplasmic membrane, generating pores that act as the mediators of pyroptosis and as direct conduits for the transport of IL-1β and IL-18 (Shi J. et al., 2015; Boucher et al., 2018), ultimately causing pyroptosis and inflammatory response (Liu X. et al., 2016). Moreover, caspase-1 results in the cleavage of pro-IL-1β and pro-IL-18 and the release of active IL-1β and IL-18 (Miao et al., 2011), which recruit inflammatory cells and further amplify the inflammatory response. The plasma membrane pores allow the flow of ions, leading to the usual equilibrium of ion gradients between the intracellular and extracellular environments; the water then enters the cell, causing swelling and dissolution (Fink and Cookson, 2006). In the noncanonical caspase-4/5/11-dependent pathway of pyroptosis, the inflammatory agents, such as bacterial lipopolysaccharides (LPS), activate caspase-4/5/11, cleave GSDMD, and release GSDMD-p30 (Kayagaki et al., 2011; Kayagaki et al., 2015), which binds to the plasma membrane and the mitochondrial membrane to kill cells by forming the pyroptotic pores (Aglietti et al., 2016). Besides GSDMD, other members of the gasdermin family, such as GSDMA, GSDMB, GSDMC, GSDME/DFNA5, and GSDMA3, have similar functions in the cytotoxicity of cell membrane disruption (Ding et al., 2016). In the absence or blocking of canonical or noncanonical pyroptosis pathway (caspase1/4/5/11-GSDMD), pyroptosis can be engaged by mechanisms such as caspase-8-GSDMD (Orning et al., 2018) and caspase-3-GSDME (Wang et al., 2017), but their contribution to pyroptosis has not been determined *in vivo*.

Although originally discovered in bacterial immunity, pyroptosis has gradually become a recognized form of RCD in biological scenarios. Pyroptosis serves as an effective antimicrobial defense for the host during infection (Aachoui et al., 2013). However, the mutations in NOD-like receptor (NLR) proteins or the persistence of sterile inflammatory stimuli can lead to excessive pyroptosis, which is harmful to the host and can increase the levels of inflammatory mediators IL-1β, IL-18, and alarmin high mobility group box-1 (HMGB-1), leading to disease if left unchecked (Bergsbaken et al., 2009).

### 3.2 Effects of Pyroptotic Cell Death and Inflammation on Atherosclerosis

**FIGURE 2:**
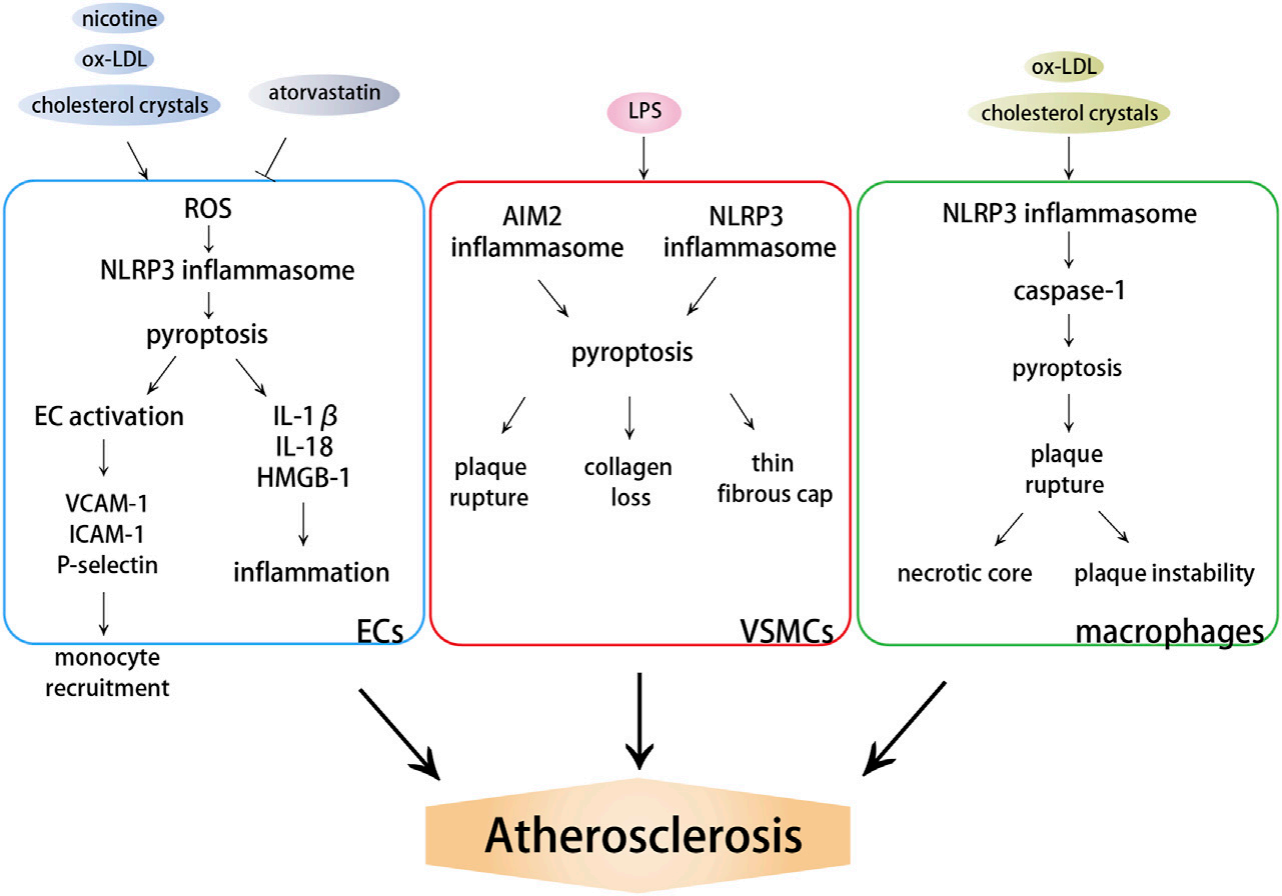
Effects of pyroptosis on atherosclerosis. DAMPs induce the activation of NLRP3 or AIM2 inflammasome, which further activate caspase-dependent pyroptosis. ECs pyroptosis triggers monocyte recruitment into the intima in early atherogenesis. VSMC pyroptosis weakens fibrous caps and contributes to pathological instability in atherosclerosis. Macrophage pyroptosis promotes the formation of necrotic core and aggravates plaque instability in advanced atherosclerotic lesions.

Inflammation is a primary response of innate immunity and is considered to be the initiator and driver of atherosclerosis. Pyroptosis, an inflammatory form of cell death, has been shown to be involved in atherosclerosis (An et al., 2019). Important functional molecules that result in pyroptosis, including NLRP3, AIM2, and caspase-1, are abundantly expressed and activated in atherosclerosis and are associated with the occurrence, accumulation, and destabilization of atherosclerotic plaques (Ozaki et al., 2015; Hoseini et al., 2018; Pan et al., 2018). The expression of NLRP1 and NLRC4 genes is upregulated in patients with atherosclerosis, and their receptors may result in the systemic alteration in inflammasome activation (Borborema et al., 2020). Caspase-11-GSDMD-mediated pyroptosis and the subsequent proinflammatory response in the pathogenesis of atherosclerosis have also been proved (Jiang M. et al., 2021). Considering that inflammasome and caspase-1 are important factors that induce pyroptosis, both the absence of inflammasome and caspase-1 deficiency alleviate atherosclerosis (Zheng et al., 2014). Caspase-1 deficiency reduces endothelial cell activation and infiltration of monocytes into intima (Gage et al., 2012; Yin et al., 2015) and attenuates ox-LDL-induced VSMC pyroptosis and IL-1β processing (Li et al., 2020), which all suppress the development of atherosclerosis. The pyroptosis of ECs, VSMCs, and monocytes/macrophages is involved in the repair and injury of the intima, but plays different roles in the progression of atherosclerosis (Figure 2).

#### 3.2.1 Endothelial Cells Pyroptosis Triggers Monocyte Recruitment Into the Intima in Early Atherogenesis

Caspase-1-dependent pyroptotic cell death of ECs and resulting increased inflammatory response are involved in atherosclerosis, especially in early atherosclerotic vascular injury. In the context of dyslipidemia and inflammation, the caspase-1–inflammasome pathway in ECs can sense the elevated lipids or DAMPs and other inflammatory mediators and activate ECs (Yin et al., 2015), which is the foremost step in the progress of atherosclerosis. The significantly increased levels of VCAM-1 and ICAM-1 caused by EC activation in ApoE^−/−^ mice fed a high-fat diet were significantly reduced after caspase-1 knockout, indicating that hyperlipidemic stimulation triggered endothelial activation and upregulated adhesion molecule expression and cytokine secretion by activating caspase-1 and promoting EC pyroptosis (Yin et al., 2015). The upregulated expression of these adhesion molecules, which might be caused by pyroptosis, triggered monocyte adhesion. Based on this, EC pyroptosis promoted the recruitment of monocytes into the intima, exposure to hyperlipidemia, uptake of lipids, differentiation into macrophages, and lipid plaque formation, thus contributing to the formation and progression of atherosclerosis (Lopez-Pastrana et al., 2015; Yin et al., 2015). However, if caspase-1 activation and pyroptosis of ECs were attenuated, monocytes were not recruited into the intima and macrophages were removed from the atherosclerotic plaques (Potteaux et al., 2011; Lopez-Pastrana et al., 2015). In addition, the inhibition of caspase-1 in dyslipidemic and inflammatory environments prevented EC pyroptosis and improved EC survival and angiogenesis mediated by vascular endothelial growth factor receptor 2, thereby preserving vascular intima integrity (Lopez-Pastrana et al., 2015). This inhibited the migration and deposition of lipids, monocytes, and SMCs into the intima and further attenuated the progression of atherosclerosis. A recent study found that the protective effects against atherosclerosis of atorvastatin, as a commonly used lipid-lowering drug in clinical practice, did not depend on cholesterol-lowering capacity, but on the ability to restrain the pyroptosis of ECs through the lncRNA NEXN-AS1/NEXN pathway (Wu L. M. et al., 2020). These results proved that caspase-1-dependent pyroptosis induced by lipid metabolism disorder played a critical role in EC activation, inflammation, and early atherosclerosis.

Except for hyperlipidemia, ECs are affected by many other factors. Smoking has long been considered a risk factor for atherosclerosis. Recent studies revealed that the pyroptosis of ECs and macrophages might be a cellular mechanism for the pro-atherosclerotic properties of nicotine with the stimulation of ROS and activation of the NLRP3 inflammasome as the upstream mediators (Wu et al., 2018; Mao et al., 2021). Moreover, trimethylamine N-oxide (TMAO), an endothelial toxic factor, promoted EC pyroptosis through the increased ROS level induced by mitochondrial respiratory complex II subunit succinate dehydrogenase B (SDHB) oxidation, thereby promoting the development of atherosclerosis (Wu P. et al., 2020). The upregulation of SDHB expression and activity could also be induced by low shear stress-mediated TET2 deletion and reduced recruitment of histone deacetylase 2 (Chen J. et al., 2021). A low shear force also could to promote EC pyroptosis through the miR-181b-5p/STAT-3 axis, causing damage to the vascular wall and tissue remodeling and hence leading to the onset and development of atherosclerosis (Xu et al., 2021). In conclusion, ECs play a central role in mediating inflammation. Hyperlipidemia and other proatherogenic risk factors can promote the activation of the inflammasome and pyroptosis of ECs, increase the secretion of adhesion factors, and significantly promote monocyte recruitment and atherosclerosis progression.

#### 3.2.2 Vascular Smooth Muscle Cell Pyroptosis Weakens Fibrous Caps and Contributes to Pathological Instability in Atherosclerosis

The death of VSMCs in atherosclerotic plaques can lead to significant thinning of fibrous caps, loss of collagen and extracellular matrix, and severe vascular inflammation, aggravating plaque instability. Except apoptosis, the pyroptosis of VSMCs is also tied up with the pathogenesis of atherosclerosis. The active pyroptotic indicators, including caspase-1 and IL-1β, overlapping with SMC marker α smooth muscle actin (α-SMA), were examined in human and mice atherosclerotic plaques, especially near the necrosis core, on the plaque surface, and in the intraplaque hemorrhage area, providing evidence that VSMC pyroptosis participated in atherosclerosis and contributed to plaque instability (Li et al., 2020). Pan et al. also reported the increased expression of GSDMD-N in ApoE^−/−^ mice. AIM2 irritates GSDMD activity and pyroptosis through the ASC-caspase-1 pathway in VSMCs, thereby accelerating the atherosclerotic plaque progression (Pan et al., 2018). LPS derived from porphyromonas gingivalis (*Pg*-LPS) caused the pyroptosis of VSMCs and instability of atherosclerotic plaques, which played an important role in the occurrence and development of atherosclerosis (Liu G. et al., 2016). However, the blockage of circular RNA serine/threonine protein phosphatase PP1-gamma catalytic subunit (circPPP1CC) relieved the *Pg*-LPS-induced pyroptosis of VSMCs and atherosclerosis by inhibiting the HMGB-1/TLR9/AIM2 pathway (Liu J. et al., 2021). In advanced plaques, the proliferation of VSMCs promoted fibrous cap formation and contributed to plaque stability (Basatemur et al., 2019). To sum up, as a form of cell death of VSMCs in plaques, pyroptosis weakened the fibrous cap and promoted plaque rupture.

#### 3.2.3 Macrophage Pyroptosis Promotes the Formation of Necrotic Core and Aggravates Plaque Instability in Advanced Atherosclerotic Lesions

Ox-LDL and cholesterol crystals could also trigger NLRP3 inflammasome activation and caspase-1 production in macrophages, leading to caspase-1-mediated pyroptosis (Duewell et al., 2010; Rajamaki et al., 2010). Rajamäki et al. reported that the silencing of NLRP3 receptors, one of the key components of the NLRP3 inflammasome, completely eliminated the cholesterol crystals-induced IL-1β secretion, thus confirming NLRP3 inflammasome as the cholesterol crystal-reactive element in macrophages (Rajamaki et al., 2010). The effect of cholesterol crystals and ox-LDL to activate the NLRP3 inflammasome depends on the destruction of lysosomes and the leakage of cathepsin, all of which promote the cleavage and activation of pro-caspase-1 and pyroptosis (Duewell et al., 2010; Karasawa and Takahashi, 2017; Grebe et al., 2018). Sequential pyroptosis occurs in response to ox-LDL or cholesterol crystals, in which one macrophage cannot digest the crystals and dies via pyroptosis. Then, another macrophage engulfs the same crystals and dies via pyroptosis repeatedly. This circulation reduces lesion cellularity, causes and exacerbates pronounced inflammation by releasing inflammatory mediators from pyroptotic cells, and promotes lesion instability (Chang et al., 2013).

Growing evidence shows that the pyroptosis of macrophages increases the risk of atherosclerotic plaque rupture, hence contributing to the formation of necrotic core and plaque instability in atherosclerotic lesions (Lu et al., 2021). Proinflammatory cytokines and mediators relying on caspase-1 in response to proatherogenic risk factors, such as ox-LDL and cholesterol crystals, trigger off pyroptosis of macrophage foam cells, resulting in the formation of lipid-rich pools, the necrotic core of plaques, in late stages of the lesion (Yan and Hansson, 2007). Additionally, the molecules including NLRP3, ASC, caspase-1, IL-1β, and IL-18 are more expressed in unstable plaques compared with stable plaques (Shi X. et al., 2015). Subsequently, Varghese et al. also demonstrated the close relationship between the upregulation of NLRP3 inflammasome-related components and plaque vulnerability, suggesting the possibility of NLRP3 inflammasome activation in promoting plaque instability (Paramel Varghese et al., 2016). Inhibiting NLRP3 expression and reducing macrophage pyroptosis could stabilize atherosclerotic plaques, which undoubtedly hinted at the potential value of blocking pyroptosis (Lin et al., 2021).

Vulnerable plaques are characterized by the accumulation of dead cells, in which up to 50% cells are macrophages. A large majority of dead cells in human atherosclerotic plaques had a typical cell lysis ultrastructure compared with apoptosis (Naghavi et al., 2003). The ruptured plaque showed strong immunoreactivity to caspase-1, but weak immunoreactivity to caspase-3, the executor caspase of apoptosis (Kolodgie et al., 2000). In plaque macrophages, the suppression of apoptosis by the targeted deletion of caspase-3 or p53, which all induced apoptosis, did not subside inflammation and atherosclerotic lesions (Merched et al., 2003). Conversely, macrophages showed the characteristics of cells undergoing pyroptosis accompanied by the increased caspase-1 activation in response to triglyceride treatment, suggesting that the cells in plaque died mainly via pyroptosis, not apoptosis (Son et al., 2013). Taken together, these data suggested that pyroptosis was involved in macrophage death in advanced atherosclerotic lesions and exacerbated inflammation through proinflammatory cytokines, ultimately leading to plaque rupture and acute coronary syndromes.

#### 3.2.4 Recent Discovery of Inhibiting Pyroptosis and Improving Atherosclerosis

Based on the aforementioned understanding, the focus on novel targets and mechanisms has increased in recent years, so as to discover new approaches to intervene pyroptosis and atherosclerosis. For example, IFN regulatory factor-1, which has been found to be upregulated in macrophages of patients with acute coronary syndrome (Guo M. et al., 2019), facilitated macrophage pyroptosis and plaque rupture in atherosclerosis by restraining circ___0029589 through promoting its m6A modification (Guo et al., 2020). Brain-derived neurotrophic factor, which is found at lower levels in patients with coronary artery disease (CAD) than in those without CAD, inhibited ox-LDL-induced NLRP3 inflammasome formation and pyroptosis of ECs through KLF2/HK1-mediated regulation of glucose metabolism and mitochondrial homeostasis (Jin et al., 2021). Aldehyde dehydrogenase 2 has been reported to suppress ox-LDL-induced NLRP3 inflammasome priming and activation by attenuating oxidative stress (Xu et al., 2020). MicroRNA-302c-3p could directly target NLRP3 and ameliorate aortic inflammation and pyroptosis in ApoE^−/−^ mice (Bai et al., 2021). Further studies are needed on the detailed mechanism of pyroptosis promoting atherosclerosis to find out better targets for atherosclerosis intervention.

## 4 The Roles of Ferroptosis in Atherosclerosis

### 4.1 Overview of Ferroptosis

Ferroptosis is an iron-dependent form of RCD resulting from the overwhelming accumulation of lipid hydroperoxides and redox imbalance, characterized by a loss of plasma membrane integrity, cytoplasmic and organelles swelling, dysmorphic shrunken mitochondria with the loss of cristae, as well as condensed and ruptured outer membrane, but the morphology of the nucleus remains unchanged (Dixon et al., 2012; Dixon and Stockwell, 2014; Galluzzi et al., 2018). Ferroptosis occurs through iron-catalyzed lipid peroxidation initiated by nonenzymatic mechanisms such as Fenton reaction, and enzymatic mechanisms including lipoxygenases (LOXs) and EGLN prolyl hydroxylases (PHD), which are enzymes responsible for lipid peroxidation and oxygen homeostasis. The increased uptake of iron by transferrin receptor 1 (TFR1) and reduced iron export by ferroportin-1 (FPN1) trigger off iron overload, in which Fenton reaction-generated ferric iron (Fe^3+^) increases ROS, activates LOXs or PHD, and results in ferroptosis (Yang and Stockwell, 2008; Gao et al., 2015; Yang et al., 2016). Therefore, the lipid peroxidation and ferroptosis can be prevented by iron chelators such as deferoxamine and dexrazoxane (Dixon et al., 2012; Friedmann Angeli et al., 2014) or by knocking down TFR1 (Gao et al., 2015), whereas decreased expression of ferritin or FPN1 has been testified to be sensitized to ferroptosis (Bao et al., 2020; Chen et al., 2020). In addition to LOXs and PHD, the two other enzymes involved in phospholipid metabolism, especially in the oxidation of specific phosphatidylethanolamine-containing polyunsaturated fatty acids (PUFAs), acyl-CoA synthetase long-chain family member 4 (ACSL4) and lysophosphatidylcholine acyltransferase 3 (LPCAT3), also contribute to ferroptosis (Dixon et al., 2015; Doll et al., 2017; Muller et al., 2017; Xiao et al., 2019). In the presence of iron, PUFAs generate lipid hydroperoxides and then produce toxic lipid free radicals, which transfer protons near PUFAs and trigger a new round of lipid oxidation reactions (Yang et al., 2016). Some radical-trapping antioxidants, such as vitamin E, ferrostatin-1, and liproxstatin-1, exhibit oxidation resistance and ferroptosis resistance (Dixon et al., 2012; Hofmans et al., 2016); they directly trap chain-carrying peroxyl radicals rather than repressing LOXs (Zilka et al., 2017).

Except for increasing iron release into the labile iron pool (LIP) mentioned earlier, impeding the activation of glutathione peroxidase 4 (GPX4) is the classical channel that acts on the occurrence of ferroptosis. As the major protective mechanism of biomembranes against lipid peroxidation by catalyzing the conversion of lipid hydroperoxides into lipid alcohols, the function of GPX4 depends on its cofactor glutathione (GSH) and the plasma membrane cystine/glutamate antiporter system x_c_^−^, which imports cystine in exchange for intracellular glutamate and generates GSH (Seiler et al., 2008; Yang et al., 2014). GPX4 can be inactivated by the deprivation of GSH through the inhibition of the system x_c_^−^, as a result of the restrained function of overloaded extracellular glutamate, erastin, sulfasalazine, sorafenib, p53, and activating transcription factor 3 (ATF3) (Yang et al., 2014; Jiang et al., 2015; Hayano et al., 2016; Wang L. et al., 2020). Buthionine-(S, R)-sulfoximine (BSO) is also able to decrease GSH synthesis (Yang et al., 2014), furthermore, some compounds that directly bind and inactivate GPX4, such as RAS-selective-lethal compound 3 (RSL3), ML162, ML210, FINO_2_, FIN56, and withaferin A, also have the ability to inhibit GPX4 and subsequently induce lipid peroxidation and ferroptosis (Weiwer et al., 2012; Friedmann Angeli et al., 2014; Yang et al., 2014; Shimada et al., 2016; Gaschler et al., 2018; Hassannia et al., 2018). In contrast, the Nrf2–Keap1 pathway has been confirmed to stimulate the system x_c_^−^ and enhance resistance to ferroptosis through Nrf2 overexpression or Keap1 downregulation (Fan et al., 2017). Moreover, several other pathways that manage to regulate the peroxide state and prevent ferroptosis are gradually being discovered and studied. A newly discovered ferroptosis-resistance factor, FSP1, has also been shown to confer protection against ferroptosis as an oxidoreductase that catalyzes the regeneration of ubiquinone (also known as coenzyme Q10, CoQ10), which can trap lipid peroxyl radicals that mediate lipid peroxidation (Bersuker et al., 2019; Doll et al., 2019). GTP cyclohydrolase-1 (GCH1) and its metabolic derivatives tetrahydrobiopterin/dihydrobiopterin (BH4/BH2) have also recently been found to protect against ferroptosis because of the effect of BH4 that selectively deters the autoxidation of phospholipids and accelerates CoQ10 (Kraft et al., 2020). These redox pathways act on the progress of ferroptosis, but their roles and in-depth mechanisms in the pathophysiology of ferroptosis need to be further explored.

Ferroptosis induces the opening of plasma membrane pores, which may be caused by the lipid peroxidation-induced conformational changes in lipid domains and plasma membrane regions (Runas et al., 2016; Agmon et al., 2018). The formation of plasma membrane pores contributes to solute exchange with the external environment, resulting in cell swelling, and then ferroptotic cells undergo rupture and death (Riegman et al., 2020). Moreover, the intercellular propagation of the ferroptosis-inducing signal, including erastin and C’ dot nanoparticles but not GPX4 inhibitor, occurs upstream of cell rupture and involves the spread of cell swelling effects through cell populations in an iron- and lipid peroxide-dependent manner (Dolma et al., 2003; Kim et al., 2016; Riegman et al., 2020). Because this wave-like propagation may cause widespread tissue damage (Katikaneni et al., 2020), ferroptosis has been demonstrated to be involved in multiple acute injury and degenerative diseases in various tissues including kidney, liver, heart, and brain (Abdalkader et al., 2018; Fang et al., 2019; Wang M. et al., 2020; Li et al., 2021), and also be a target in the treatment of cancers (Badgley et al., 2020).

### 4.2 Effects of Ferroptosis on Atherosclerosis

**FIGURE 3:**
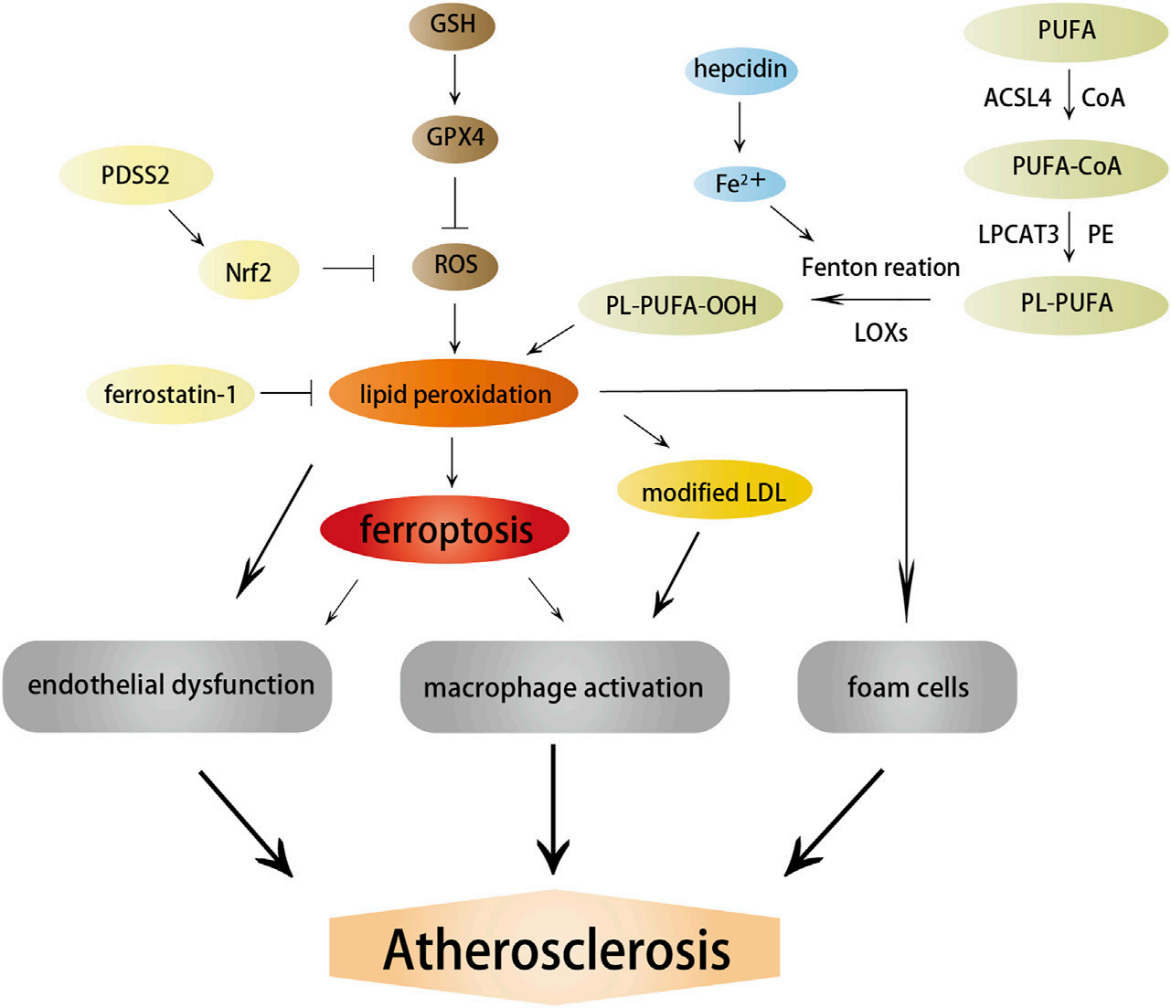
Effects of ferroptosis on atherosclerosis. Ferroptosis and lipid peroxidation induce endothelial dysfunction, macrophage activation, and foam cell formation, contributing to the generation of atherosclerosis. ACSL4, LOXs, and hepcidin promote atherosclerosis by inducing lipid peroxidation and iron overload; in contrast, PDSS2 and ferrostatin-1 can inhibit ferroptosis and atherosclerosis by suppressing lipid peroxidation.

Ferroptosis is a nonapoptotic form of RCD driven by abnormal iron metabolism and lipid peroxidation, which are related to the pathogenesis of atherosclerosis (Vinchi et al., 2020) (Figure 3). Nontransferrin-bound serum iron-induced iron overload triggers off ROS production and stimulates monocyte recruitment, leading to vascular oxidative stress and plaque formation (Vinchi et al., 2020). Lipid peroxidation, intraplaque hemorrhages, and iron deposition, which are hallmarks of advanced plaques (Martinet et al., 2019), are also considered as indirect evidence for the onset of ferroptosis. Therefore, it is reasonable to believe that ferroptosis may participate in the occurrence and development of atherosclerosis. Indeed, a recent study confirmed this hypothesis and revealed that the inhibition of ferroptosis alleviated atherosclerosis by reducing lipid peroxidation and endothelial dysfunction in mouse aortic ECs (Bai T. et al., 2020).

Studies on the pathways and regulatory factors related to ferroptosis can also reflect the correlation between ferroptosis and atherosclerosis. GPX4 is responsible for transforming lipid hydroperoxides into nontoxic lipid alcohols, which can effectively abate the damage caused by oxidized lipids to vascular cells. A decreased GPX level has been demonstrated in atherosclerosis. Guo et al. found that the overexpression of GPX4 that could suppress ferroptosis reduced lipid peroxidation and alleviated atherosclerotic lesions in ApoE^−/−^ mice (Guo et al., 2008). Moreover, the positive correlation between the severity of atherosclerosis and ACSL4 also implied that ferroptosis regulated the occurrence and progression of atherosclerosis (Zhou et al., 2021). Increased expression of ferroptosis-associated LOXs promoted atherosclerosis, while inhibiting 12/15-LOX significantly diminished ox-LDL subendothelial deposition and weakened the development of atherosclerosis (Li C. et al., 2018). Yang et al. recently found that prenyldiphosphate synthase subunit 2 (PDSS2), which was a key enzyme for the synthesis of CoQ10, could alleviate ferroptosis and atherosclerosis by activating antioxidant Nrf2 and suppressing ROS release and iron content (Yang et al., 2021). Radical-trapping antioxidant ferrostatin-1 has also been found to ameliorate iron overload, lipid peroxidation, and upregulation of ROS production induced by high glucose and high lipids, thereby effectively reducing ferroptosis and atherosclerosis (Meng Z. et al., 2021).

Ferritin-mediated iron homeostasis is instrumental in the cardiovascular system (Vinokur et al., 2016), and excessively increased ferritin levels can promote early atherosclerosis and its related complications (Sung et al., 2012). A clinical study found that circulating ferritin levels were independently associated with the changes in carotid intima-media thickness, suggesting that it could be used to monitor the risk of atherosclerosis (Prats-Puig et al., 2016). TFR1 promoted iron absorption and ferritin synthesis, and was found to accumulate significantly in the nuclear region of many foam cells, contributing to the development and rupture of atherosclerotic plaques (Li et al., 2008). Hepcidin, another hormone modulator of iron homeostasis, served as a catalyst for ferroptosis by increasing intracellular iron levels (Nemeth et al., 2004) and enhanced inflammatory atherosclerosis by inducing iron deposition (Habib et al., 2015). Therefore, the factors related to iron metabolism are important targets to the resistance of atherosclerosis, but whether they protect against atherosclerosis by affecting ferroptosis needs further exploration.

## 5 Possible Association Between Autophagy and Pyroptosis in Atherosclerosis

**FIGURE 4:**
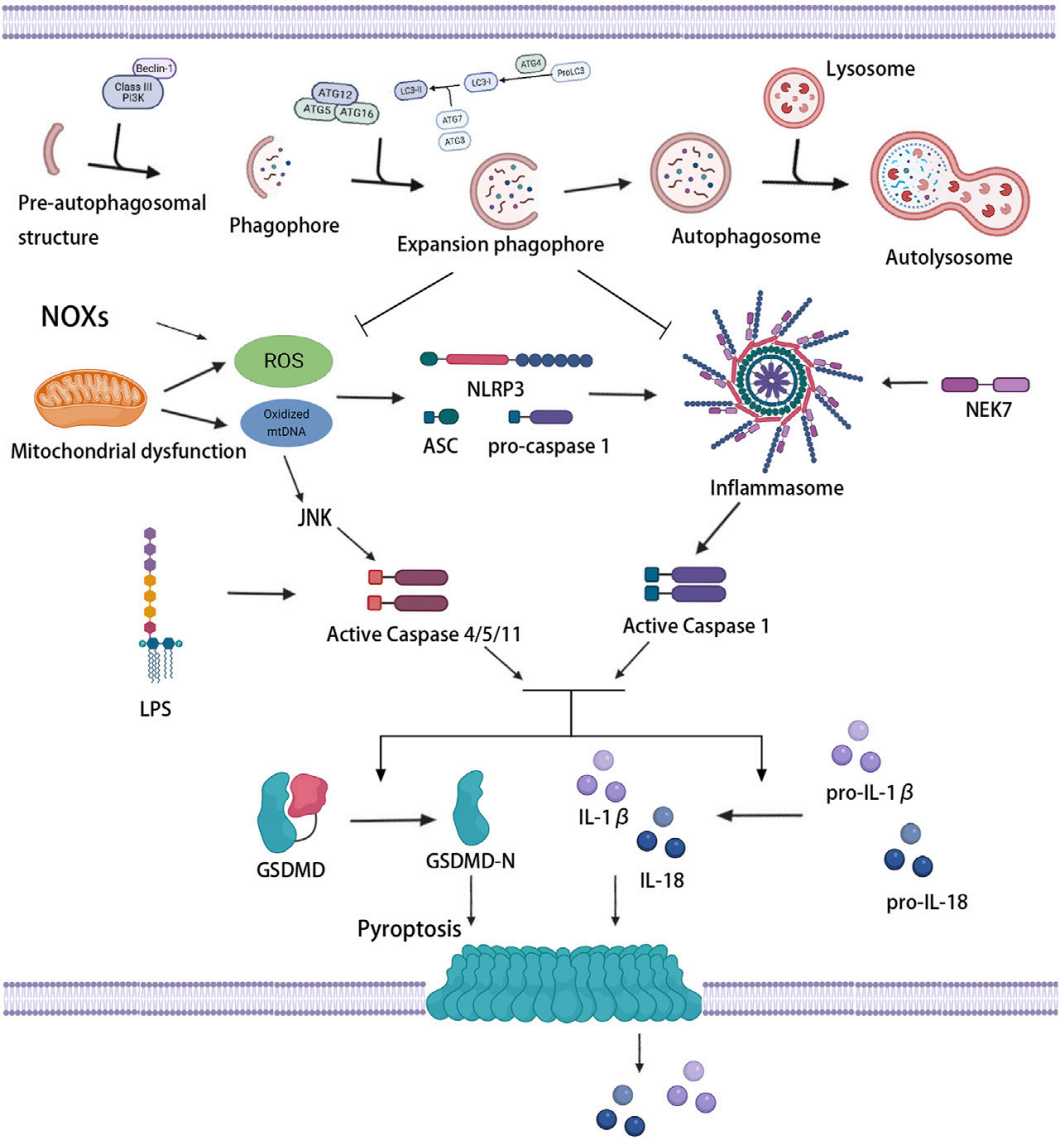
Association between autophagy and pyroptosis. Mitochondrial dysfunction and NOXs induce the massive production of ROS and stimulate the activation of inflammasome and caspase-1. LPS and oxidized mtDNA induce caspase-4/5/11 activation. Activated caspase-1/4/5/11 cleaves GSDMD, induces pyroptotic cell death, and promotes the release of IL-1β and IL-18. Mild oxidative stress triggers off autophagy, which inhibits inflammasome signaling and ROS production, thus protecting against pyroptosis.

As shown in Figure 4, the level of autophagy has a regulatory effect on pyroptosis. The effect has been verified and applied in treating tumors, infectious diseases, and cardiovascular and cerebrovascular diseases. In recent years, great strides have been made in the mutual regulation of autophagy and pyroptosis in atherosclerosis. Inducing an increase in autophagic activity in vulnerable atherosclerotic plaques restrains NLRP3 inflammasome activation and secretion of inflammatory cytokines, thus ameliorating inflammation and attenuating lipid deposition and pyroptosis (Peng et al., 2018; Cong et al., 2020). In contrast, repressing autophagy aggravates NLRP3 inflammasome activation and pyroptosis (Jiang et al., 2018). As previously mentioned, autophagy defects or autophagy deficiency are normally present in the progression of atherosclerosis. Autophagy deficiency has been shown to lead to atherosclerosis through multiple complementary mechanisms, including overactivation of the inflammasome, accumulation of cytotoxic protein aggregates, and impaired lipid degradation, which are all triggers of pyroptosis.

### 5.1 Autophagy, Pyroptosis and Oxidative Stress

Various signaling pathways of pyroptosis regulated by autophagy have always been a hot spot for research, especially oxidative stress signaling pathways. Oxidative stress occurs when the production of pro-oxidants or ROS exceeds the capacity of endogenous antioxidants. ROS, which are produced mainly by mitochondria and nicotinamide adenine dinucleotide phosphate (NADPH) oxidase (NOX), refer to the general term of oxygen-containing free radicals and peroxides that can easily form free radicals related to oxygen metabolism in the organism. Wang et al. found that NOX-mediated accumulation of ROS which stimulated by hypoxia promoted pyroptosis, meanwhile, autophagic activity was lessened in this process, suggesting that severe oxidative stress induced the upregulation of pyroptosis, as well as the inhibition of autophagy (Wang S. et al., 2019).

The increase in the levels of intracellular ROS give rise to pyroptosis through the execution of caspase (Li et al., 2015). The activation of caspase depends on the inflammasome, and oxidative stress is an initial signal that induces inflammasome activation and influences the priming phase of inflammasomes (Bauernfeind et al., 2011; Swanson et al., 2019). The massive accumulation of ROS can trigger off the activation of the NLRP3 inflammasome and then induce caspase-1-dependent pyroptosis (Qiu et al., 2017), which may be related to an ROS-sensitive NLRP3 ligand, thioredoxin-interacting protein (TXNIP/VDUP1) (Zhou et al., 2010). Ox-LDL, which is abundant in atherosclerotic lesions, has been certified to increase the activity of ROS and promote the activation of inflammasome and caspase-1, leading to pyroptosis (Ding et al., 2013b; Lin et al., 2013). Furthermore, increased mitochondria-derived ROS and oxidation of liberated mtDNA from damaged or dysfunctional mitochondria can also drive the assembly and activation of the NLRP3 inflammasome (Zhou et al., 2011; Zhong et al., 2018). Oxidized mtDNA was found to bind to NLRP3 directly, promoting inflammasome function and subsequent caspase-1 activation (Shimada et al., 2012). In contrast, the downregulation of NLRP3 inflammasome activation by ROS scavenger could effectively suppress caspase-1 cleavage, IL-1β production, and pyroptosis (Chen et al., 2016; Long et al., 2020). Antioxidants extracellular superoxide dismutase and catalase also showed the ability to prevent caspase-1 activation and pyroptosis (Xi et al., 2016). In addition, c-Jun N-terminal kinase (JNK) signaling played a role in the noncanonical caspase-4/5/11-dependent pathway of pyroptosis (Abe and Morrell, 2016). ROS activated JNK and transferred it into the nucleus, promoted the expression of caspase-11, and initiated pyroptosis (Lupfer et al., 2014). These results suggested that the activation of inflammasomes associated with oxidative stress induced the initiation of pyroptosis, and the extent of pyroptosis seemed to increase with the increase in inflammasome stimulation.

ROS mediates NLRP3 inflammasome activation, but also upregulates autophagy to inhibit the excessive activation of NLPR3 inflammasome (Jiang L. et al., 2021). For autophagy, ROS is one of the upstream modulator (Szumiel, 2011). Under acute or chronic oxidative stress, autophagy is increased or decreased, respectively (Mitter et al., 2014). Mild oxidative stress triggers some cellular survival and repair mechanisms, including autophagy pathways. Indeed, autophagy increases the bioavailability of NO and restrains oxidative stress and vascular inflammation (LaRocca et al., 2012). Autophagy can also selectively clear dysfunctional mitochondria which leads to massive production of ROS, thus inhibiting ROS accumulation and subsequent inflammasome activation (Li Q. et al., 2018; Lin et al., 2019). In addition, as a mitochondrial outer membrane protein that directly interacted with LC3, NIX was found to promote the degradation and removal of excess mitochondria and ROS by regulating autophagy, thus inhibiting ox-LDL-induced pyroptosis of macrophages in atherosclerosis (Peng et al., 2020). However, tumor necrosis factor receptor-associated factor 3 (TRAF3) has been reported to form a complex with TRAF2 and cIAP1 and mediate ubiquitin and degradation of ULK1, in which mitochondrial ROS production and pyroptosis were promoted (Shen et al., 2020). Therefore, ROS-activated autophagic machinery may represent a negative feedback mechanism that limits ROS-regulated caspase-1 activation through removing ROS-damaged organelles and proteins. Nevertheless, conversely, severe oxidative stress produces a large number of damaged proteins, which may lead to an overload of the autophagosome system, resulting in a reduced autophagic activity. Wang et al. reported that a decreased miR-103 level in oxidatively stressed ECs suppresses the interaction between autophagosomes and lysosomes, restraining the end-stage of autophagy, promoting the accumulation of inflammatory mediators, and thus increasing the occurrence of pyroptosis (Wang Y. et al., 2020).

Accordingly, considering that ROS can promote inflammasome activity but autophagy can suppress ROS production, the suppression of autophagy on inflammasome activity and pyroptosis are related to oxidative stress. ROS blockade with subsequently suppressed inflammasome activity by autophagy is one of the important mechanisms to antagonize pyroptosis. An important feedback network exists between autophagy, NF-κB signaling pathway, and pyroptosis (Gao Y. et al., 2020). Wang et al. reported that enhanced autophagy prevented pyroptotic cell death by eliminating excess ROS and negatively mediating the nuclear translocation of NF-κB P65 and NLRP3 inflammasome activation (Wang X. et al., 2019). Li et al. found that adrenomedullin protected the steroidogenic functions of Leydig cells against pyroptosis by activating autophagy through the ROS-AMPK-mTOR axis (Li et al., 2019). Autophagy activation reversed the expression of pyroptosis-related proteins by downregulating the level of IL-13 and inhibiting the Janus kinase (JAK)-1/signal transducers and activators of transcription (STAT)-1 signaling pathway (Gao C. et al., 2020). Nrf2/ARE, one of the key pathways involved in oxidative stress, can regulate the expression of scavenger receptor-B in the macrophages of ApoE^−/−^ Nrf2^+/+^ mice, stimulate the rapid uptake of ox-LDL by macrophages, and promote the formation of atherosclerotic plaques (Bozaykut et al., 2014). A recent study further revealed that activation of the Nrf2/ARE pathway might be influenced by autophagy. The blockage of autophagy promoted the pyroptosis of ox-LDL-treated macrophages through the malignant activation between p62 and Nrf2/ARE pathway, which might provide a novel therapeutic target for atherosclerosis (Liu J. et al., 2021). Thereout, autophagy suppresses pyroptosis partly due to its regulation of oxidative stress, while devastating oxidative stress induces pyroptosis by inhibiting autophagy.

### 5.2 Autophagy, Pyroptosis and Inflammation

Atherosclerosis is a chronic inflammatory disease, and pyroptosis can release the cytoplasmic contents of dead host cells, thus providing an effective signal to initiate an inflammatory cascade, which exacerbates vascular inflammation and atherosclerosis (Man et al., 2017). Some proinflammatory cytokines are expressed in human atherosclerotic lesions, particularly in relation to infiltrating macrophages. Macrophages are considered as the primary sources of IL-1β and IL-18, which are two major substrates of caspase-1 and are mainly responsible for caspase-1-mediated pyroptosis in atherosclerosis, promoting plaque destruction and arterial thrombosis through the release of chemokines, matrix metalloproteinases, and proteases (Dinarello, 2011). Vromman et al. found that IL-1β inhibition promoted the tilt of monocytes toward a less inflammatory state during atherosclerosis and reduced the size of established atherosclerosis (Vromman et al., 2019). Moreover, IL-1β inhibition could recede the expression of VCAM-1 and monocyte chemotactic protein-1 (MCP-1), thus preventing the recruitment of monocytes or macrophages into the intima and significantly reducing atherosclerotic lesions (Kirii et al., 2003). The Canakinumab Anti-inflammatory Thrombosis Outcome Study trial involving 10,061 patients with previous myocardial infarction also revealed that anti-inflammatory therapy targeting the IL-1β innate immune pathway resulted in a significant reduction in the recurrence rate of cardiovascular events, independent of a decrease in lipid levels (Ridker et al., 2017). These results established a pivotal role for IL-1β in driving inflammation during atherogenesis and the evolution of advanced atheroma. Similarly, IL-18 has been found to promote the development of atherosclerosis by promoting lipoprotein oxidation, macrophage-derived foam cell formation, and immune cell activation (Bahrami et al., 2021). Even so, Gomez et al. demonstrated the atheroprotective effects of IL-1β in advanced atherosclerotic lesions. The results highlighted the importance of promoting inflammatory resolution, while the excessive repression of inflammation disrupted the formation and maintenance of SMC- and collagen-rich fibrous caps, leading to atherosclerotic plaque instability (Gomez et al., 2018). Thus, the excessive release of IL-1β and IL-18 attributed to pyroptosis is one of the factors that aggravate atherosclerosis, but meanwhile, the over-inhibition of inflammation also leads to plaque instability.

Pyroptosis and the release of these inflammatory factors requires the activation of inflammasome. Recent evidence has revealed a complex interplay between autophagy and inflammasome. Autophagy may be accompanied by the activation of inflammasomes. *Pseudomonas aeruginosa* can activate the NLRP3 inflammasome to trigger macrophage autophagy so as to escape intracellular killing (Deng et al., 2016). In addition, as mentioned earlier, mitochondria are involved in inflammasome activation; mitophagy is also stimulated in this process and regulates inflammasome activity by removing damaged mitochondria, thereby providing an important negative feedback mechanism (Green et al., 2011; Nakahira et al., 2011; Zhong et al., 2016). Autophagy moderates inflammation by eliminating active inflammasomes; however, lack of autophagy leads to the opposite effect and thus exacerbates inflammation. Resolvin D2, which is an innate suppressor of inflammation, has been found to promote the degradation of NLRP3 in an autophagy dependent manner; the suppression of autophagy could reverse the inhibition of NLRP3 inflammasome (Cao et al., 2021). LPS-dependent inflammasomes are activated in macrophages whose autophagy is blocked by the genetic ablation of the autophagy regulators ATG16L1 or ATG7, but not in wild-type macrophages, suggesting that autophagy generally suppresses inflammasome activation induced by LPS (Saitoh et al., 2008). The NLRP3 inflammasome can also be activated by oxidative or apoptosis-induced mtDNA, which is released during autophagy repression (Sollberger et al., 2015). Impaired CMA has also been proven to increase NLRP3 inflammasome activation and secretion of IL-1β, promoting vascular inflammation and atherosclerosis (Qiao et al., 2021). Therefore, autophagy can negatively regulate pyroptosis by restraining the activation of the inflammasome and caspase-1/GSDMD pathway (Wang X. et al., 2020). A clinical trial found that excessive inflammation and the pyroptosis of vascular ECs triggered off atherosclerosis in postmenopausal women, which could be ameliorated with estrogen treatment by alleviating EC pyroptosis through estrogen receptor α-mediated activation of autophagy (Meng Q. et al., 2021). For the mechanism underlying autophagy-dependent inflammation inhibition, it has been suggested that autophagy typically restrains inflammasome signaling and IL-1β production by directing ubiquitinated inflammasome complexes and pro-IL-1β to lysosomes for degradation (Katsnelson et al., 2016; Kimura et al., 2017). In other words, the inflammasome itself may serve as a target for autophagic degradation, thereby facilitating the resolution of the inflammatory responses.

In spite of an activation effect on autophagy, the NLRP3 inflammasome has also been found to suppress protective autophagy through the E2/ERβ/AMPK/mTOR pathway or through caspase-1-mediated cleavage of the TLR adaptor (Lai et al., 2018; Wei et al., 2019). Moreover, the triggering of pyroptosis releases a large number of proinflammatory factors, further aggravating the inflammatory response, which in turn aggravates the autophagy defect; the two synergistically exacerbate atherosclerosis. For example, a high level of myeloid cells trigger receptors 1 (TREM-1) and a low level of Sirt6 are associated with increased risk for all-cause mortality and major adverse cardiovascular events. Also, TREM-1 negatively regulates the expression of Sirt6. TREM-1-mediated pyroptosis may underlie the proatherogenic effects of ox-LDL, which may be limited by Sirt6-induced EC autophagy (Zi et al., 2019). In conclusion, autophagy activation could inhibit pyroptosis and excessive inflammation, thereby improving atherosclerosis and reducing the incidence of adverse cardiovascular events, which associated with decreased inflammasome activity.

### 5.3 Autophagy, Pyroptosis and Lipid Accumulation

Dyslipidemia, which involves pathologically elevated concentrations of cholesterol and other lipids, is a major risk factor for atherosclerotic diseases (Kopin and Lowenstein, 2017). The inflammasome may be a potential target of atherosclerosis induced by abnormal lipid metabolism. The NLRP3 inflammasome is positively correlated with the severity and prognosis of atherosclerosis in patients with acute coronary syndrome (Afrasyab et al., 2016). When the LDLR^−/−^ mice were transplanted with NLRP3-deficient, ASC-deficient, or IL-1α/β-deficient bone marrow and fed a high cholesterol diet, they had significantly reduced early atherosclerosis and inflammasome-dependent IL-18 levels (Duewell et al., 2010), suggesting that the deficiency of the NLRP3 inflammasome significantly protected proatherogenic mice from disease. Given that pyroptosis is an inflammasome-dependent form of cell death, possible lipotoxic mediators including ox-LDL, cholesterol crystals, saturated fatty acids, and palmitic acid were found to induce pyroptosis in ECs (Hu et al., 2018), which involve mitochondrial injury, oxidative stress, activation of JNK and release of DAMPs. LDL entered the arterial wall and was transformed into ox-LDL due to changes in endothelial permeability and extracellular matrix composition located under the endothelium (Tabas et al., 2007). Ox-LDL might induce EC pyroptosis and promote the development of atherosclerosis by regulating the miR-125a-5p/ten-eleven translocation 2 (TET2) pathway, resulting in mitochondrial dysfunction, increased ROS generation, and NF-κB nuclear transposition that triggered the activation of the NLRP3 inflammasome and inflammatory response (Zhaolin et al., 2019). The effect of ox-LDL on the NLRP3 inflammasome also depended on the upregulated expression of mixed lineage kinase domain-like protein (Wu Q. et al., 2020). Cholesterol crystals, a key pathological marker of atherosclerotic plaque vulnerability, which were observed in human atherosclerotic lesions as they progressed from fatty streaks to more advanced lesions (Katz et al., 1976), could induce ROS production, NLRP3 inflammasome activation, and ultimately pyroptosis of ECs (Yang et al., 2020). The major lipid component of the plasma membrane, lysophosphatidylcholine, was also proved to elicit the activation of the inflammasome and pyroptosis though potassium efflux and lysosomal damage in both ECs and monocytes; this process was involved in the maintenance of inflammation in tissues adjacent to blood vessels and the pathological process of atherosclerosis (Correa et al., 2019). These results suggested that dyslipidemia was an important factor to induce pyroptosis, and inflammasome activation might be an important link between lipid metabolism and inflammation in atherosclerotic lesions. Therefore, regulation of lipid metabolism and inhibition of lipid overaccumulation are important ways to inhibit inflammasome activation and pyroptosis in atherosclerosis.

Besides pyroptosis, lipid accumulation may also lead to defects in autophagy. In advanced human carotid atherosclerosis, accompanied by lipid accumulation, the levels of autophagic proteins ATG5 and LC3β were decreased, while the levels of dysfunctional autophagy markers SQSTM1/p62 and ubiquitin were increased (Li et al., 2016). Defects in autophagic machinery might occur during the formation and maturation of autophagosomes or during the fusion of autophagosomes with lysosomes or lysosomal-mediated degradation. It was widely considered that an autophagy defect in atherosclerosis was attributable to defects in the lysosomal-dependent pathway. Abundant cholesterol crystals in atherosclerotic plaques jeopardized the lysosomal membrane, consequently impeding the autophagy process (Duewell et al., 2010). These findings suggested that lysosomal-mediated cytoplasmic degradation might be restrained in advanced atherosclerosis, rather than the initiation of autophagy itself. Therefore, autophagy may still be actuated in response to the autophagy activator in the plaques, such as ROS and oxidative lipids, but the process becomes dysfunctional in the second stage of lysosomal-dependent degradation.

Endothelial autophagy represents an important mechanism to regulate excess, exogenous lipids and limit lipid accumulation in the vascular wall. Oxidized and natural LDL promotes the formation of autophagosomes, while excess natural or modified LDL appears to be engulfed in the autophagic structure, which is intercepted by endothelial-specific ATG7 knockdown, showing an increase in lipid deposition (Torisu et al., 2016). Recently, Kim et al. found that endothelial lipid accumulation in atherosclerosis is caused by autophagy-related lysosomal dysfunction (Kim et al., 2021). Moreover, autophagy deficiency disturbs the degradation of CAV1, which interacts with caveolae associated protein 1 (CAVIN1) and LC3B, and is used to form more caveolae in the cell membrane, contributing to LDL transcytosis across ECs (Bai X. et al., 2020). Therefore, the absence of autophagy of ECs is responsible for the prolonged retention of oxidized and natural LDL and increased atherosclerotic burden. Some researchers reported that ox-LDL in atherosclerosis induced EC autophagy, accompanied by apoptosis and increased monolayer permeability; however, when ox-LDL-mediated autophagy was partly inhibited, apoptosis and monolayer permeability were further increased (Wei et al., 2013; Wang Y. et al., 2019).

A recent study demonstrated that activation of the P2RY12 receptor reduced cholesterol efflux and promoted the formation of VSMC-derived foam cells by blocking autophagy in advanced atherosclerosis, suggesting that autophagy is essential in maintaining lipophagic flux and cholesterol homeostasis in VSMCs (Pi et al., 2021). Similarly, as mentioned earlier, intact autophagic machinery is also essential in limiting lipid uptake by macrophages. C1q/tumor necrosis factor-related protein 13 can diminish ox-LDL uptake, retention, and foam cell formation, meanwhile reducing inflammatory responses and lesion areas, which is attributed to the enhanced autophagy in macrophages and accelerated autophagy-lysosome-dependent degradation of CD36 (Wang C. et al., 2019). Gustafsson and colleagues also found that perilipin-2, whose Pro251 variant had a negative correlation with intima-media thickness at baseline and over 30 months of follow-up, initiated a feed-forward cycle in which liver-X-receptor and autophagy activate each other, thus influencing the susceptibility to atherosclerosis through the increased activation of autophagy and stimulation of cholesterol efflux (Saliba-Gustafsson et al., 2019). Based on this, we have reason to believe that enhancing autophagy can inhibit pyroptosis by regulating lipid accumulation in vascular cells, although there is no clear literature confirming this viewpoint.

## 6 Possible Association Between Autophagy and Ferroptosis in Atherosclerosis

### 6.1 Regulation of Autophagy on Ferroptosis

**FIGURE 5:**
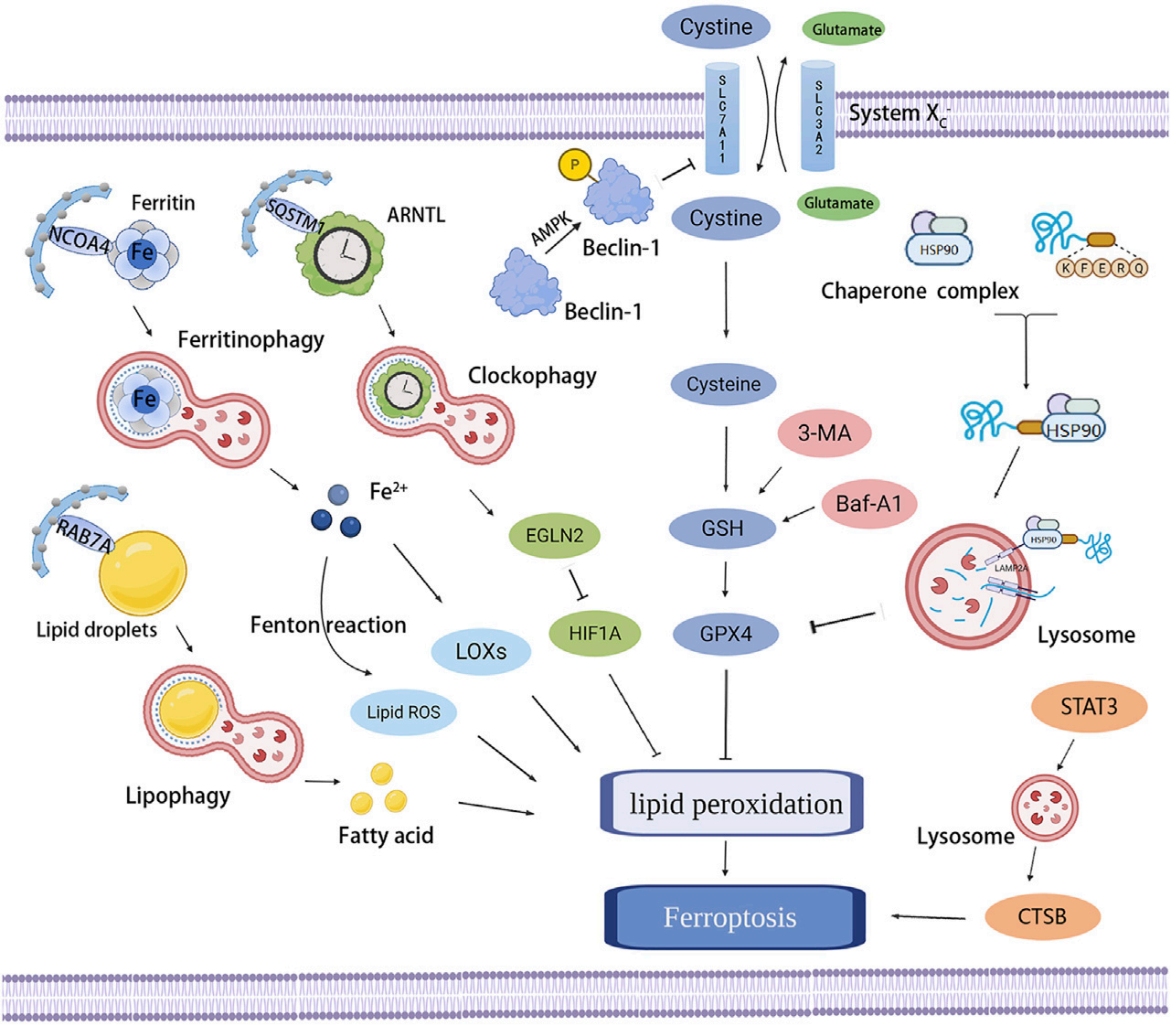
Association between autophagy and ferroptosis. Several types of selective autophagy, including ferritinophagy, lipophagy, clockophagy, and CMA, promote lipid peroxidation and ferroptosis by promoting the degradation of ferritin, lipid droplets, ARNTL, and GPX4, respectively. Beclin-1 is phosphorylated by AMPK and then facilitates ferroptosis by binding and blocking the activity of system x_c_^−^. Autophagy inhibitors (e.g., 3-MA, Baf-A1) can prevent GSH depletion-dependent ferroptosis. STAT3-mediated CTSB expression and lysosomal cell death promote ferroptosis.

It has shown that the knockout or knockdown of ATG5 and ATG7 prevents erastin-induced ferroptosis by diminishing intracellular iron levels and lipid peroxidation (Hou et al., 2016), suggesting that autophagy may play a critical role in the progress of ferroptosis. As shown in Figure 5, Song et al. found that the autophagy regulator Beclin-1 was phosphorylated by AMPK and translocated to the plasma membrane, which ultimately facilitated ferroptosis through binding and blocking the activity of system x_c_^−^ (Song et al., 2018). Autophagy also decreases intracellular GSH levels, and thus, autophagy inhibitors such as 3-MA and Baf-A1 can prevent GSH depletion-dependent ferroptosis (Desideri et al., 2012; Sun Y. et al., 2018). Since lysosomes recycle endogenous iron sources like ferritin and mitochondria, it participates in ferroptosis by influencing cellular iron equilibria and ROS generation (Torii et al., 2016). STAT3 positively regulated ferroptosis by mediating cathepsin B (CTSB) expression. Erastin increased lysosomal membrane permeability and subsequent lysosomal cell death, which could be inhibited by the pharmacological blockade of cathepsin activity. The repression of lysosomal activity and the release of acidic hydrolases into the cytoplasm could restrain ferroptosis (Torii et al., 2016; Gao et al., 2018).

Apart from nonselective autophagy, the excessive activation of selective autophagy that recognizes specific targets and recruits corresponding receptors for subsequent lysosomal degradation mediates ferroptosis. Excess iron can prompt the overproduction of mitochondrial ROS and actuate ferroptosis. Ferritin is the main iron storage protein, which inhibits oxidative stress caused by Fe^2+^-mediated Fenton reaction and maintains iron homeostasis. Excessive degradation of ferritin can significantly increase intracellular unstable iron levels and enhance sensitivity to ferroptosis (Dufrusine et al., 2019). Ferritin-targeted autophagy, known as ferritinophagy, induces ferroptosis by promoting the release of iron stored in ferritin to the LIP and also lipid peroxidation (Tang et al., 2018). An increasing number of studies reported that activating ferritinophagy promoted ferroptosis by degrading ferritin and inducing TFR1 expression (Du et al., 2019; Sui et al., 2019; Sui et al., 2021), while ferritinophagy deficiency led to cell survival during ferroptosis (Masaldan et al., 2018; Zhang Z. et al., 2020). Ferritinophagy is mediated by nuclear receptor coactivator 4 (NCOA4), which is a cargo receptor for the degradation of ferritin. NCOA4 increases the amount of intracellular iron by binding to FTH1 in autophagosomes and sending the autophagosomes to lysosomes to degrade ferritin (Mancias et al., 2014). Therefore, silencing NCOA4 prevents ferroptosis by abrogating the accumulation of iron and ROS, while the overexpression of NCOA4 increases ferritin degradation and promotes ferroptosis (Gao et al., 2016; Qin et al., 2021). Furthermore, Park et al. confirmed that ferritinophagy was indeed actuated by erastin-induced ROS, providing evidence that the activation of ferritinophagy induced by the ferroptosis inducer was necessary for the initiation of ferroptosis (Park and Chung, 2019).

In addition to ferritinophagy, Yang et al. first revealed that clockophagy, which was a novel mode of selective autophagy, could degrade the core circadian clock protein aryl hydrocarbon receptor nuclear translocator-like protein 1 (ARNTL) depending on the cargo receptor sequestosme 1 (SQSTM1), and favored lipid peroxidation and ferroptosis resulting from type II ferroptosis activators such as RSL3 and FIN56 (Yang et al., 2019). ARNTL could directly repress transcriptional factor egl-9 family hypoxia inducible factor 2 (EGLN2) expression and subsequently increase the level of prosurvival factor hypoxia-inducible factor 1 subunit α (HIF1A), while HIF1A suppressed ferroptosis by regulating lipid metabolism and facilitating the storage of lipids in droplets (Bai et al., 2019). Thus, the clockophagy-mediated degradation of ARNTL induced ferroptosis through the activation of lipid peroxidation. In addition, lipid droplets or increased lipid storage protected against ferroptosis (Kamili et al., 2015). Lipophagy was found to promote ferroptosis by degrading lipid droplets into free fatty acids that then trigger off mitochondrial oxidation, which could be suppressed by the knockout of ATG5 (Bai et al., 2019). In lipophagy, as the key mediator of transporting multivesicular bodies and lysosomes to the lipid droplet surface, RAB7A (a member of the RAS oncogene family) promoted lipid peroxidation and ferroptosis (Schroeder et al., 2015; Bai et al., 2019). Moreover, CMA is a selective autophagy that uses molecular chaperone such as heat shock proteins (HSPs) to stimulate the direct translocation of certain cytoplasmic proteins across the lysosomal membrane by recognizing specific amino acid sequences. It has been shown that autophagy of HSP70 stabilizes lysosomes and inhibits ferroptosis by promoting the transformation of lysosomal redox-active iron into a non-redox-active form (Kurz and Brunk, 2009). HSP member 5 (HSPA5) protected against GPX4 degradation and lipid peroxidation by bounding GPX4 (Zhu et al., 2017). However, Wu et al. found that ferroptosis activators facilitated the degradation of GPX4 protein by increasing the levels of lysosome-associated membrane protein 2a (LAMP2A) and inducing CMA, besides, the inhibition of CMA stabilizes GPX4 and reduces ferroptotic cell death through HSP90 inhibitor (Wu Z. et al., 2019), supporting that HSPs-mediated CMA plays a critical role in ferroptosis. In a nutshell, these findings overturned the previous conclusion that ferroptosis was non-autophagic cell death and confirmed that ferroptosis was an autophagy-mediated form of cell death. The initiation of ferroptosis required autophagy, and ferroptosis was an autophagic process.

### 6.2 Effect of Ferroptosis on Inflammation

Like pyroptosis, ferroptosis can also promote sterile inflammation and the development of inflammatory diseases by releasing DAMPs (Gong et al., 2020), in which GSH and GPX4 play an important role. As the most abundant antioxidant, GSH can buffer increasing ROS and protect cell from inflammation-associated damage (Mak et al., 2017). Moreover, GPX4 inhibited inflammatory response by suppressing arachidonic acid oxidation and NF-κB pathway activation, meanwhile reducing ROS production (Li C. et al., 2018). In contrast, the disruption of GPX4 could upregulate the expression of 12-lipoxygenase and cyclooxygenase 1, thereby triggering an inflammatory response (Chen et al., 2003). Kang et al. have found that the deficiency of GPX4 in myeloid cells increased the production of caspase-1/11-mediated GSDMD and promoted pyroptosis, which also could promote inflammatory response by releasing plenty of inflammatory factors (Kang et al., 2018).

Ferroptosis-associated peroxidation of LDL induces endothelial dysfunction and macrophages activation; besides, lipid peroxidation increases ROS, decreases NO, and promotes the formation of foam cells, all of these contributing to atherosclerosis (Yu et al., 2021). A recent study found that activation of Sirt1 inhibited excess iron-induced ferroptosis of foam cells through autophagy, providing a novel therapeutic target for atherosclerosis (Su et al., 2021). However, as mentioned earlier, autophagy induces ferroptosis through a variety of pathways, but the role of these regulatory mechanisms of autophagy in ferroptosis of vascular cells in atherosclerosis remains unclear. Furthermore, although we have manifested a correlation between atherosclerosis and ferroptosis, of course, which mainly involved the ferroptosis-related factors including iron overload and redox pathways, whether ferroptosis is active or passive, in other words, whether it is an automatic response to a stimulus that disrupts metabolic homeostasis or it is a stimulus that directly disrupts the homeostasis is still controversial. Therefore, further studies are needed to explore the exact relationship between ferroptosis and pathophysiological conditions of atherosclerosis, and also the interaction between autophagy and ferroptosis in atherosclerosis.

## 7 Conclusions and Future Perspectives

Oxidative stress plays a key role in the pathogenesis of numerous types of vascular diseases including atherosclerosis. Once exceeding the capacity of the cellular reductase mechanisms, an overwhelming concentration of ROS triggers off direct or indirect functional damage to different molecules, such as proteins, lipids, and nucleic acids, eventually leading to cell death, which unfortunately leads to, rather than ameliorating, atherosclerosis and plaque instability. ROS-induced inflammasome activation and lipid peroxidation are important features of atherosclerosis, therefore, pyroptosis and ferroptosis as nexus linking oxidative stress, inflammation and lipid metabolism are inevitable to play important roles in the pathogenesis of atherosclerosis. Autophagy is a promising pathway for maintaining plaque cell survival and function. Increasing evidence has shown the crosstalk between autophagy, pyroptosis, and ferroptosis, but whether these different modes of RCD can be integrated into a complete regulatory network remains to be explored.

In atherosclerosis, excessive oxidative stress, inflammation, and lipid peroxidation lead to autophagy dysfunction, pyroptosis, and ferroptosis. The regulation of autophagy, pyroptosis, and ferroptosis is thought to be the potential therapeutic option of atherosclerosis. However, the current studies still have significant limitations. Firstly, atherosclerosis is a complex pathological process involving multiple cells, and the results of a single cell type or a single cell process can hardly explain the actual disease. It is a pity that although we have found clues from separate studies, the specific mechanism of mutual regulation of multiple RCDs and the mutual influence of multiple vascular cells on atherosclerosis has not been implemented. Second, although some studies have used animal models, a large number of experiments *in vivo* are still urgently needed, especially clinical experiments.

In summary, the abnormal states of autophagy, pyroptosis, and ferroptosis in vascular cells including ECs, VSMCs and macrophages associated with oxidative stress, inflammation, and lipid peroxidation are important pathogenesis of atherosclerosis. However, their regulatory mechanisms of atherosclerosis need to be further explored, especially the interaction between multiple RCDs. Therefore, further researches on RCD will develop a broader field for exploring the pathogenesis of atherosclerosis and provide a better clinical treatment for atherosclerosis.
